# Neutrophil-Associated Central Nervous System Inflammation in Tuberculous Meningitis Immune Reconstitution Inflammatory Syndrome

**DOI:** 10.1093/cid/ciu641

**Published:** 2014-08-08

**Authors:** Suzaan Marais, Katalin A. Wilkinson, Maia Lesosky, Anna K. Coussens, Armin Deffur, Dominique J. Pepper, Charlotte Schutz, Zahiera Ismail, Graeme Meintjes, Robert J. Wilkinson

**Affiliations:** 1 Clinical Infectious Diseases Research Initiative, Institute of Infectious Disease and Molecular Medicine; 2 Department of Medicine, University of Cape Town, South Africa; 3 Division of Mycobacterial Research, MRC National Institute for Medical Research; 4 Department of Medicine, Imperial College London, United Kingdom

**Keywords:** tuberculosis, HIV, therapy-complications, antiretroviral therapy

## Abstract

***Background.*** The immunopathogenesis of tuberculosis-associated immune reconstitution inflammatory syndrome (IRIS) remains incompletely understood, and we know of only 1 disease site-specific study of the underlying immunology; we recently showed that *Mycobacterium tuberculosis* culture positivity and increased neutrophils in the cerebrospinal fluid (CSF) of patients with tuberculous meningitis (TBM) are associated with TBM-IRIS. In this study we investigated inflammatory mediators at the disease site in patients with TBM-IRIS.

***Methods.*** We performed lumbar puncture at 3–5 time points in human immunodeficiency virus (HIV)–infected patients with TBM (n = 34), including at TBM diagnosis, at initiation of antiretroviral therapy (ART) (day 14), 14 days after ART initiation, at presentation of TBM-IRIS, and 14 days thereafter. We determined the concentrations of 40 mediators in CSF (33 paired with blood) with Luminex or enzyme-linked immunosorbent assays. Findings were compared between patients who developed TBM-IRIS (n = 16) and those who did not (TBM-non-IRIS; n = 18).

***Results.*** At TBM diagnosis and 2 weeks after ART initiation, TBM-IRIS was associated with severe, compartmentalized inflammation in the CSF, with elevated concentrations of cytokines, chemokines, neutrophil-associated mediators, and matrix metalloproteinases, compared with TBM-non-IRIS. Patients with TBM-non-IRIS whose CSF cultures were positive for *M. tuberculosis* at TBM diagnosis (n = 6) showed inflammatory responses similar to those seen in patients with TBM-IRIS at both time points. However, at 2 weeks after ART initiation, S100A8/A9 was significantly increased in patients with TBM-IRIS, compared with patients with TBM-non-IRIS whose cultures were positive at baseline.

***Conclusions.*** A high baseline *M. tuberculosis* antigen load drives an inflammatory response that manifests clinically as TBM-IRIS in most, but not all, patients with TBM. Neutrophils and their mediators, especially S100A8/A9, are closely associated with the central nervous system inflammation that characterizes TBM-IRIS.

## Abstract (precis)

Tuberculous meningitis immune reconstitution inflammatory syndrome (TBM-IRIS) is characterized by severe, compartmentalized cerebral inflammation, involving mediators of innate and adaptive immune responses. A high baseline cerebrospinal fluid bacillary load predisposes to recurrent inflammation during antiretroviral therapy, manifesting as TBM-IRIS.

Because of the rapid scale-up of antiretroviral therapy (ART) programs, tuberculosis-associated immune reconstitution inflammatory syndrome (TB-IRIS) is a significant contributor to the healthcare burden in high TB/HIV coinfection settings [1–3]. Paradoxical TB-IRIS presents as clinical deterioration after the initiation of ART in patients who have improved or stabilized with tuberculosis treatment before ART initiation [4]. This deterioration occurs in the context of a rapid restoration of *Mycobacterium tuberculosis*–specific immune responses. Neurological TB-IRIS is the most severe form of TB-IRIS, with an associated mortality of 13%–75% [3, 5–7], compared with the 3.2% estimate for all forms of paradoxical TB-IRIS combined [8].

Although recent studies have advanced understanding of TB-IRIS through the identification of several cellular, immunological, and genetic factors associated with the syndrome, the immunopathogenesis remains incompletely defined, and no diagnostic test exists [9]. Thus far, explorations of the immune response in TB-IRIS pathogenesis have been of those measured in blood, which is likely an incomplete representation of the immune response in affected tissue—for example, the central nervous system (CNS).

In this study, we investigated inflammatory mediators in cerebrospinal fluid (CSF; 40 mediators) and blood (33 mediators) from human immunodeficiency virus (HIV)–infected patients with tuberculous meningitis (TBM) who started ART during tuberculosis treatment. We compared serial findings between patients who did and those who did not develop paradoxical TBM-IRIS. The clinical findings of this cohort were described elsewhere [3]; here we focus on the immunological aspects of TBM-IRIS pathogenesis.

## METHODS

### Setting and Participants

We conducted a prospective, observational study at a public sector hospital in Cape Town, South Africa, recruiting ART-naive HIV-infected adults who presented with TBM during a 20-month period. The details of the clinical methods have been published elsewhere [3]. Both TBM and paradoxical TBM-IRIS were diagnosed according to published case definitions [4, 10]; ART-naive HIV-infected patients without meningitis who presented with symptoms and/or signs necessitating a lumbar puncture were enrolled as control participants. The University of Cape Town Human Research Ethics Committee approved the study, and written informed consent was obtained from all patients or their relatives (Supplementary Data).

### Procedure

**Figure 1.**
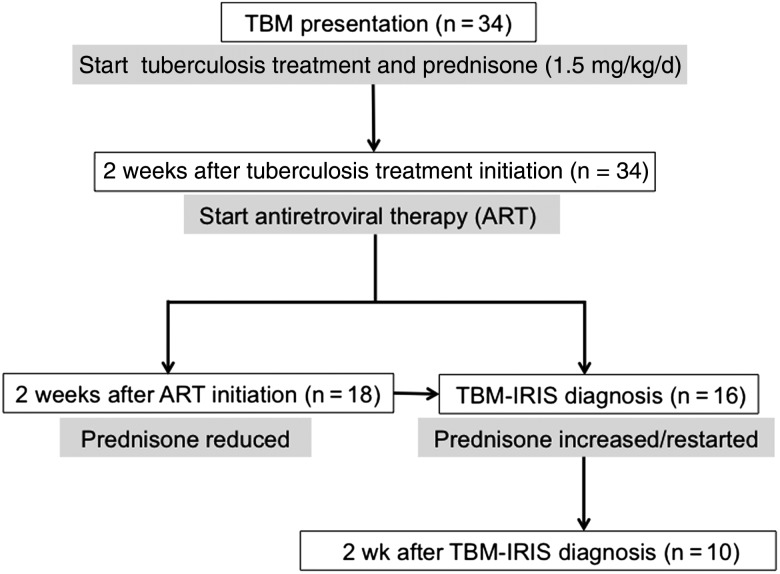
Flow diagram of time points when lumbar puncture and phlebotomy were performed in patients with tuberculous meningitis (TBM). Drug interventions are indicated at each time point (*gray boxes*). Procedures were performed at a minimum of 3 time points: (1) TBM diagnosis, (2) antiretroviral treatment (ART) initiation, and (3) 2 weeks after ART initiation or TBM immune reconstitution inflammatory syndrome (IRIS) presentation, whichever occurred first. Patients in whom TBM-IRIS developed later than 2 weeks after ART initiation underwent repeated procedures at TBM-IRIS presentation. Unless lumbar puncture was contraindicated, procedures were repeated in patients with TBM-IRIS 2 weeks after TBM-IRIS presentation. At the start of the study, the first-line ART regimen for patients receiving tuberculosis treatment in South Africa was stavudine, lamivudine, and efavirenz (started in 10 patients [63%] with TBM-IRIS and 10 [56%] with TBM without IRIS [TBM-non-IRIS]). Later during the study, tenofovir replaced stavudine, according to revised national guidelines (started in 4 patients [25%] with TBM-IRIS and 5 [28%] with TBM-non-IRIS). Five patients (2 [13%] with TBM-IRIS and 3 [17%] with TBM-non-IRIS), in whom these regimens were contraindicated, received zidovudine, lamivudine, and efavirenz.

Paired CSF and blood samples were collected from patients with TBM at 3–5 time points, shown in Figure 1. Samples were collected at 1 time point from control participants. Samples were stored at −80°C and analyzed in batches as detailed below. The primary outcome measure was CSF mediator concentrations, at the time of TBM-IRIS presentation in patients who developed TBM-IRIS and 2 weeks after ART initiation in those who did not. The 2 weeks after ART initiation time point in patients with TBM without IRIS (TBM-non-IRIS) was based on the median time (14 days after ART initiation) reported for TB-IRIS development [4]. Secondary analyses included comparisons of CSF and blood findings between (1) the combined TBM group and controls, (2) patients with TBM-IRIS and TBM-non-IRIS at TBM diagnosis and ART initiation, and (3) time points within TBM-IRIS and TBM-non-IRIS groups.

### Luminex Multiplex and Enzyme-Linked Immunosorbent Assays

Mediators analyzed in CSF and serum samples with Luminex multiplex assays included tumor necrosis factor (TNF), interferon (IFN)-γ, interleukin (IL)-2, interleukin 4, interleukin 10, interleukin 13, interleukin 1β, interleukin 6 (IL-6), interleukin 12p40 (IL-12p40), interleukin 17 (IL-17), IFN-α2, CC chemokine ligand (CCL) 2, CCL3, CCL4, CXC chemokine ligand (CXCL) 1–3, CXCL8, granulocyte colony-stimulating factor (G-CSF) and granulocyte-macrophage colony-stimulating factor (GM-CSF). Matrix metalloproteinase (MMP) 1, 2, 3, 7, 9, 10, 12, and 13, and tissue inhibitors of MMP (TIMP) 1 and 2 were analyzed with Luminex multiplex assays in CSF and plasma. Mediators measured with enzyme-linked immunosorbent assays in CSF and serum samples included IL-12p70, interleukin 17A (IL-17A), interleukin 21, 22, and 23, and CXCL10. The CSF samples were also analyzed with enzyme-linked immunosorbent assays for interleukin 18 (IL-18) and neutrophil-associated mediators: cathepsin G, lipocalin 2, LL-37, human neutrophil peptides 1–3, complement 5a (C5a), and S100A8/A9 (Supplementary Data).

### Statistical Analysis

Statistical analysis was performed using GraphPad Prism (version 5; GraphPad) and R (version 3.0) software [11]. We compared variables between groups using Wilcoxon rank sum tests. Within groups, we compared variables between time points and between blood and CSF compartments, using Wilcoxon matched-pairs tests. Correlations were estimated using the Kendall τ coefficient. We performed unsupervised agglomerative hierarchical clustering on mediators in CSF samples, using complete linkage and Euclidean distance measures. We assessed cluster-wise stability with 2 resampling schemes (bootstrap and subsetting; 100 resampling runs), and we computed the Jaccard similarities [12, 13]. Throughout the analyses, we used an unadjusted *P* value <.05 as a nominal threshold for statistical significance, except for the correlation analysis, which provides *P* values adjusted for the false discovery rate (Benjamini and Hochberg) [14]. Owing to the large number of statistical tests, our *P* values should be used for guidance in interpretation rather than finality (see Supplementary Data for details on multivariate analysis).

## RESULTS

### Demographic and Clinical Results

The demographic characteristics and baseline blood results of patients with TBM (n = 34) and controls (n = 14) are presented in Table 1. Sixteen patients with TBM developed TBM-IRIS, a median of 14 days (IQR, 4–20 days) after starting ART, and 18 did not [3]. Of note, 15 of 16 patients with TBM-IRIS and 6 of 18 with TBM-non-IRIS had *M. tuberculosis* cultured from CSF samples at TBM diagnosis (*P* < .001). There were no significant differences in CD4 cell counts and plasma HIV loads between patients with TBM and controls (Table 1). The CD4 counts and plasma and CSF HIV loads were reported elsewhere for these TBM-IRIS and TBM-non-IRIS groups and did not differ significantly between groups either at baseline or during follow-up [3].

**Table 1.** Baseline Characteristics of Patients Presenting With Tuberculous Meningitis and Controls Without Meningitis

Characteristic	Median Value (IQR)^a^		*P* Value^b^
	Patients With TBM (n = 34)	Controls (n = 14)^c^	
Age, y	33 (28–44)	34 (27–38)	.95
Female, No. (%)	15 (44)	9 (64)	.34
Body mass index	20.0 (18.3–22.7)	20.5 (19.9–27.3)	.17
Blood values			
Sodium, mmol/L	129 (123–131)	135 (133–137)	<.001
Hemoglobin, g/dL	11.4 (8.8–13.1)	12.4 (10.8–13.4)	.10
C-reactive protein, mg/L	40 (6–78)	5 (1–8)	.005
CD4 count, cells/µL	113 (69–199)	129 (75–180)	.91
HIV load, log_10_	5.46 (4.82–5.89)	4.87 (4.44–5.44)	.06
CSF values			
Lymphocyte count, ×10^6^/L	177 (87–339)	6 (2–13)	<.001
Neutrophil count, ×10^6^/L	20 (2–42)	0 (0–0)	<.001
Protein, g/L	1.94 (1.29–3.06)	0.51 (0.38–0.87)	<.001
Glucose, CSF/blood ratio	0.30 (0.17–0.5)	0.53 (0.5–0.74)	<.001

Abbreviations: CSF, cerebrospinal fluid; HIV, human immunodeficiency virus; IQR, interquartile range; TBM, tuberculous meningitis.

^a^ Values represent medians (IQRs) unless otherwise specified.

^b^ *P* values were calculated for comparisons between groups, using the Wilcoxon rank sum test for continuous variables and the Fisher exact test for categorical variables. Differences were considered statistically significant at *P* < .05.

^c^ Diagnoses in controls included HIV-associated psychosis (n = 4), tension headache (n = 4), generalized tonic-clonic seizures (n = 2), meningioma (n = 1), stroke (n = 1), depression (n = 1), and HIV-associated neurocognitive disorder (n = 1).

### CSF Mediator Concentrations in Patients With TBM and Controls

Interleukin 2, 4, 13, 21, and 23, IL-12p70, MMP-12, and MMP-13 were excluded from all analyses because of minimal or no detection in CSF and blood samples (Supplementary Data). Compared with controls with no meningitis at presentation, patients with TBM had significantly higher (*P* < .05) CSF concentrations of 28 of 32 mediators; only CCL2 and IL-17 levels were similar, and IL-18 and C5a had medians equal to 0 in both groups (Supplementary Table 2). Conversely, concentrations of only 6 of 25 mediators in blood differed between groups; IFN-γ, IL-6, CXCL8, CXCL10, and MMP-3 concentrations were higher (*P* < .05), and CCL2 concentrations were lower (*P* = .045) in patients with TBM.

### Highly Compartmentalized Inflammatory Responses in TBM With or Without IRIS Development

**Figure 2.**
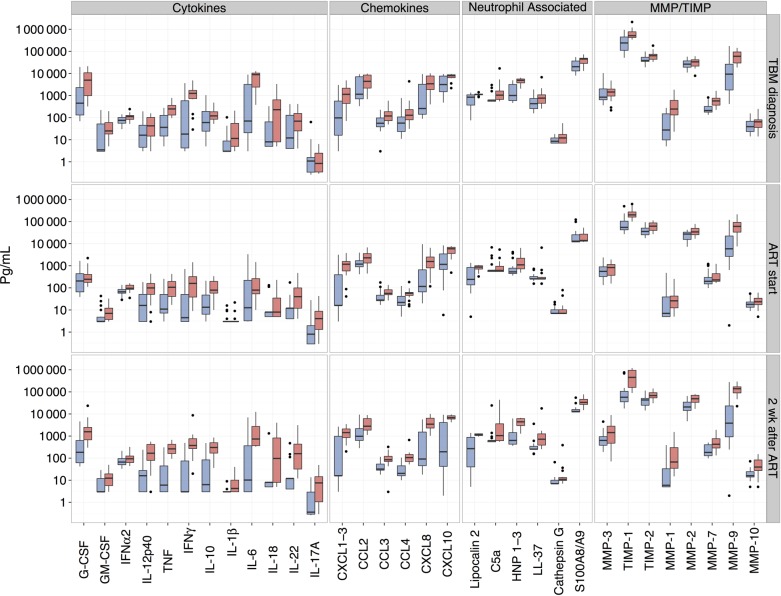
Box plots of mediator concentrations over time in cerebrospinal fluid of patients who developed tuberculous meningitis (TBM) immune reconstitution inflammatory syndrome (IRIS) (*red*) and those who did not (*blue*). The assay limits of detection have been substituted for 0 values. The left y-axis is a log_10_ scale, and the right y-axis indicates time points of sample collection. Within graphs, boxes with horizontal lines represent interquartile ranges (IQR) and medians, and vertical line represent 95% confidence intervals. Data points for outliers (≥1.5 × IQR) are included. (See Supplementary Tables 3–5 for *P* values of analyses between groups.) For patients with TBM-IRIS, the “2 wk after antiretroviral therapy (ART)” time point indicates findings at TBM-IRIS presentation, which occurred a median of 14 days (interquartile range, 4–20 days) after initiation of ART. Concentrations of all mediators were measured as picograms per milliliter, with the exception of cathepsin G, which was measured in units per milliliter. Abbreviations: C5a, complement 5a; CCL, CC chemokine ligand; CXCL, CXC chemokine ligand; G-CSF, granulocyte colony-stimulating factor; GM-CSF, granulocyte-macrophage colony-stimulating factor; HNP, human neutrophil peptide; IFN, interferon; IL, interleukin; MMP, metalloproteinase; TIMP, tissue inhibitor of MMP; TNF, tumor necrosis factor.

Within the TBM group at the time of TBM diagnosis, cytokine and chemokine concentrations were significantly higher in CSF than in blood samples, with the exception of IL-12p40, which showed comparable levels in blood and CSF, and CXCL1-3, which showed a higher trend (*P* = .06) in blood (Supplementary Table 2). Conversely, levels of MMP-1–3, MMP-7, and MMP-10 were significantly higher in blood than in CSF samples, and only MMP-9 and its inhibitor TIMP-1 were increased in CSF relative to blood (*P* < .001). Within the control group, blood cytokine, chemokine, MMP, and TIMP concentrations were either similar to or higher than CSF concentrations, with the exceptions of G-CSF, IFN-α2, CCL2, CCL3, and CXCL8 concentrations, which were higher in CSF. Consistent with the combined TBM group, a highly compartmentalized inflammatory response was seen in both subgroups when the TBM group was divided into those who did and those who did not subsequently develop TBM-IRIS (Figure 2, Supplementary Figure 1, and Supplementary Tables 3–5).

### CSF Mediator Concentrations and Dynamic Changes Over Time in TBM-IRIS Compared With TBM-Non-IRIS

**Figure 3.**
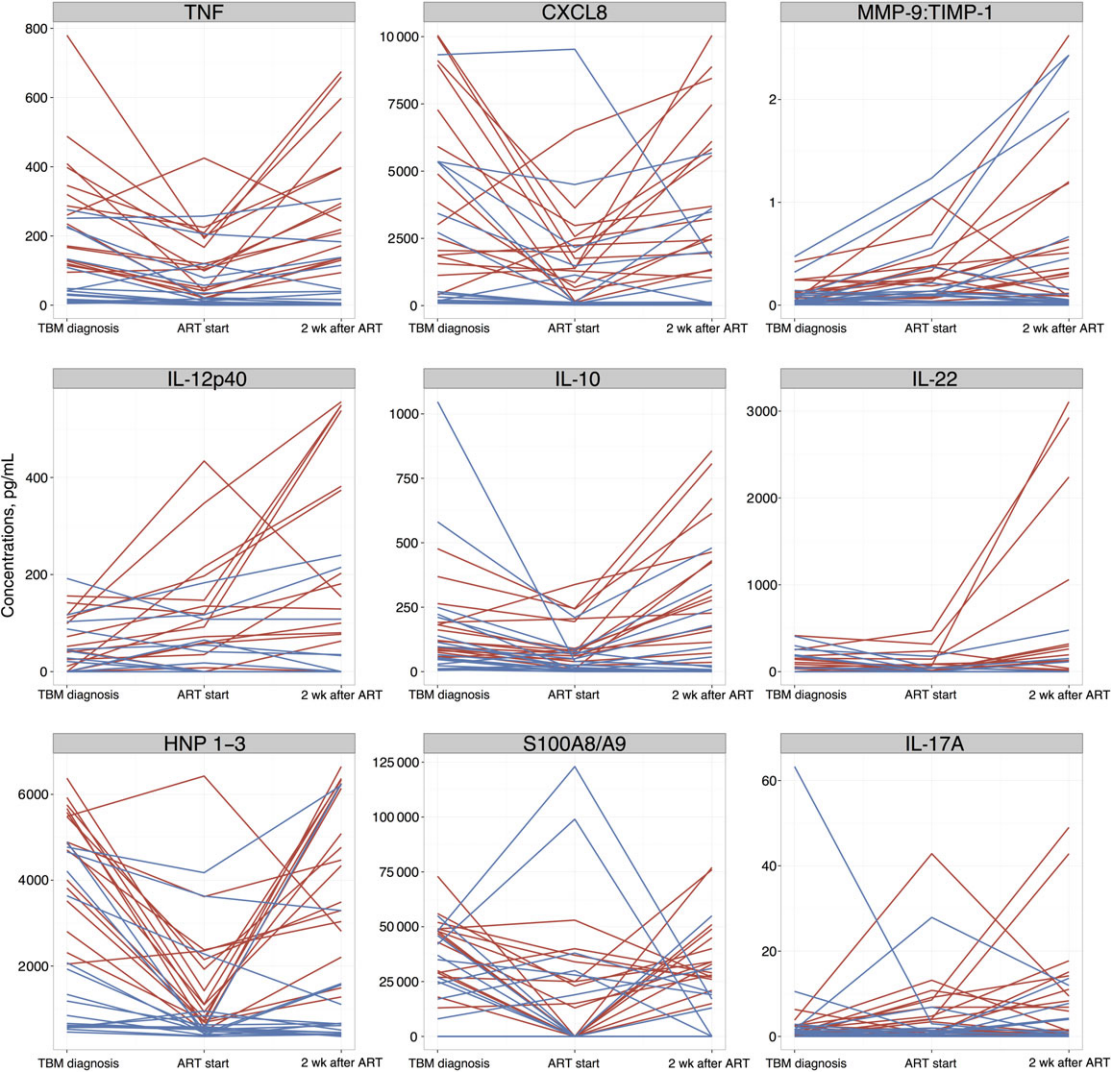
Representative examples of changes over time of cerebrospinal fluid (CSF) mediators in patients who developed tuberculous meningitis (TBM) immune reconstitution inflammatory syndrome (IRIS) (n = 16) (*red*) and those who did not (n = 18) (*blue*). For patients with TBM-IRIS, the “2 wk after ART [antiretroviral therapy]” time point indicates findings at TBM-IRIS presentation. Some patients with TBM without IRIS (TBM-non-IRIS) showed an increase in mediator concentration and the ratio of metalloproteinase (MMP) 9 to tissue inhibitor of MMP tissue inhibitor of MMP (TIMP) 1 after starting ART; these patients had CSF cultures positive for *Mycobacterium tuberculosis* at TBM diagnosis. Abbreviations: CXCL, CXC chemokine ligand; HNP, human neutrophil peptide; IL, interleukin; TNF, tumor necrosis factor.

At the time of TBM diagnosis, patients who subsequently developed TBM-IRIS, compared with those who did not, showed significantly increased CSF T-helper 1 cells (IFN-γ and IL-18) and other proinflammatory cytokines (TNF, IL-6, interleukin 1β, G-CSF, and GM-CSF), IFN-α2, chemokines (CCL2–4, CXCL1-3, CXCL8, and CXCL 10), neutrophil-associated mediators (human neutrophil peptides 1–3, lipocalin 2, C5a, and S100A8/A9), MMP-1, MMP-7, MMP-10, TIMP-1 and TIMP-2 (Figure 2 and Supplementary Table 3). Figure 3 and Supplementary Table 6 show the changes in CSF mediator concentrations over time for patients with TBM-IRIS or TBM-non-IRIS. In both groups there was a significant decrease in most mediators tested in CSF during tuberculosis treatment before initiation of ART. However, IL-12p40 and IL-17A showed a significant increase from baseline in patients with TBM-IRIS, but not those with TBM-non-IRIS, before initiation of ART.

At TBM-IRIS presentation (compared with 2 weeks after ART initiation in patients with TBM-non-IRIS), patients with TBM-IRIS showed significantly higher CSF concentrations for almost all mediators (31 of 32), except IFN-α2 (Figure 2 and Supplementary Table 5). Antiretroviral therapy was associated with marked rises in CSF inflammatory mediator concentrations in patients with TBM-IRIS, despite adjunctive corticosteroid therapy (13 of 16 patients were still receiving prednisone), such that concentrations reached similar or higher levels at TBM-IRIS presentation, approximately 2 weeks after ART initiation, compared with those at TBM presentation (Supplementary Table 6 *A*). Conversely, in TBM-non-IRIS no significant CSF changes were observed between the start of ART and 2 weeks thereafter (Supplementary Table 6 *B*). Moreover, comparing findings between samples taken 2 weeks after starting ART to those from TBM diagnosis in TBM-non-IRIS, a significant decrease was noted in CSF concentrations of G-CSF, GM-CSF, CCL3, CCL4, TNF, IFN-γ, IL-6, CXCL8, CXCL10, lipocalin 2, S100A8/A9, MMP-10, and TIMP-1. This suggests that the rise in CSF mediators observed in TBM-IRIS 2 weeks after ART reflected IRIS development, not simply the effects of ART prescription.

Samples taken 2 weeks after TBM-IRIS presentation were available for analysis in 10 of 16 patients with TBM-IRIS; death (n = 1) or contraindications to lumbar puncture (n = 5) precluded CSF sampling in the others. Compared with mediator concentrations at TBM-IRIS presentation (when prednisone was restarted or the dose increased), the only significant changes in CSF mediators were decreased concentrations of G-CSF (*P* = .04) and LL-37 (*P* = .02), 2 weeks after TBM-IRIS.

### Modest Between-Group Differences in Blood Compared With CSF Samples

At TBM diagnosis, blood concentrations were similar between TBM-IRIS and TBM-non-IRIS with the exception of IFN-α2 (*P* = .03), CXCL8 (*P* = .005), and MMP-1 (*P* = .04) concentrations, which were higher in patients with TBM-IRIS (Supplementary Figure 1 and Supplementary Table 3). The CXCL8 concentration remained elevated in TBM-IRIS at the start of ART (*P* = .02; Supplementary Figure 1 and Supplementary Table 4). Two weeks after the start of ART, concentrations of TNF, IFN-γ, CCL4, and MMP-7 (.01 <*P* <.05 for all) and CXCL8 and CXCL10 (.001 <*P* <.01 for both) were elevated in blood samples from the TBM-IRIS group compared with the TBM-non-IRIS group, and TIMP-2 was higher in the TBM-non-IRIS group (*P* = .046; Supplementary Figure 1 and Supplementary Table 5).

### CSF Neutrophil Counts and S100A8/A9 in Patients With TBM-IRIS and Culture-Positive Patients With TBM-Non-IRIS

**Figure 4.**
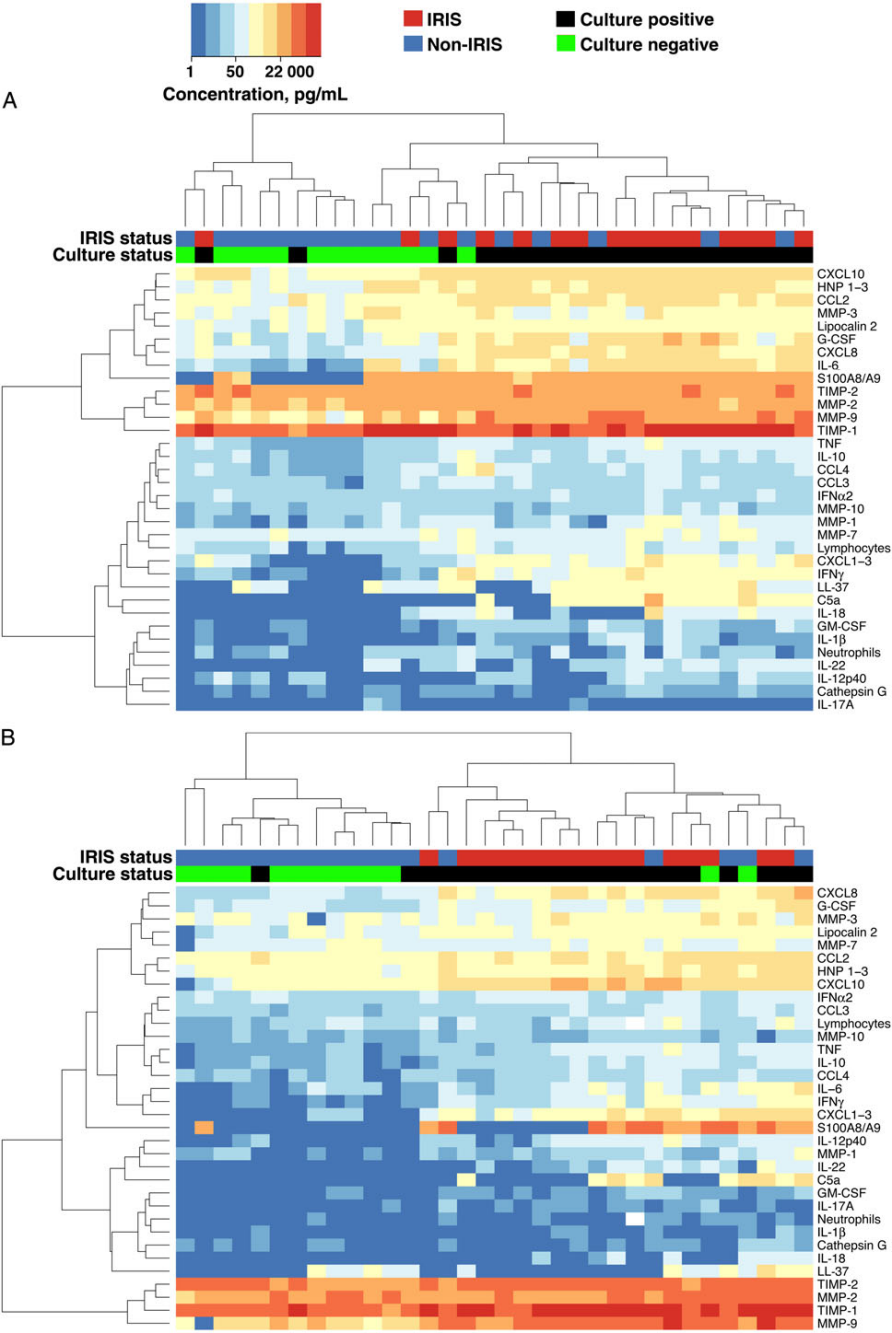

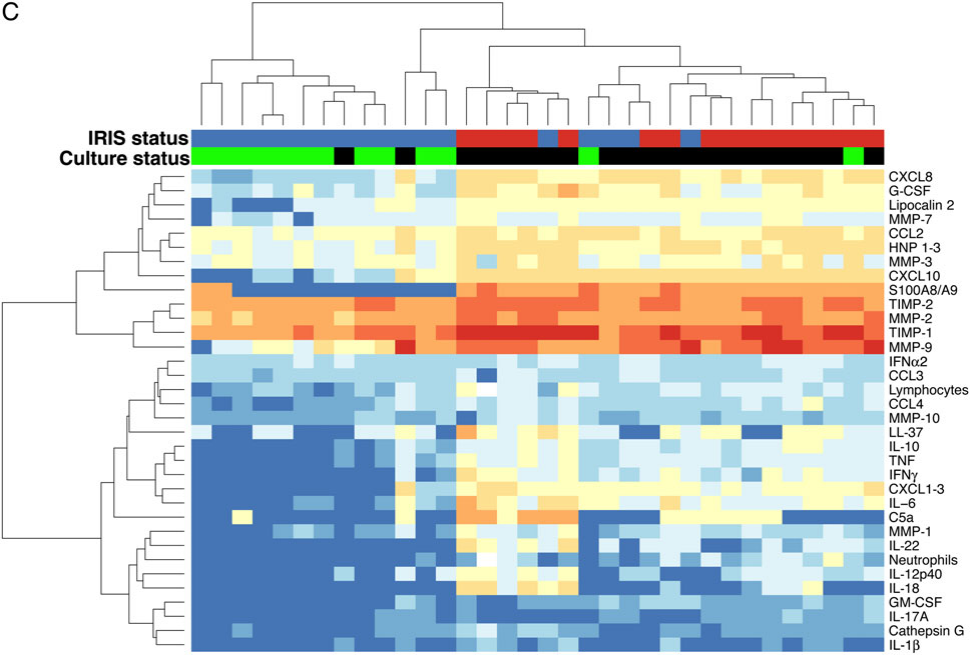
Unsupervised hierarchical clustering of tuberculous meningitis (TBM) at TBM diagnosis (*A*), initiation of antiretroviral therapy (ART) (*B*), and TBM immune reconstitution inflammatory syndrome (IRIS) presentation or 2 weeks after ART initiation (patients with non-TBM-IRIS) (*C*). IRIS status indicates patients who developed TBM-IRIS (*red*) and those who did not (*blue*). Culture status indicates cerebrospinal fluid cultures positive (*black*) or negative (*green*) for *Mycobacterium tuberculosis* at TBM diagnosis. Values plotted are natural log + 1. Corresponding concentrations in picograms per milliliter are indicated on the color key. Concentrations of all mediators were measured in picograms per milliliter, with the exception of cathepsin G, measured in units per milliliter; neutrophils and lymphocytes were measured as cells × 10^6^ per liter. Abbreviations: C5a, complement 5a; CCL, CC chemokine ligand; CXCL, CXC chemokine ligand; G-CSF, granulocyte colony-stimulating factor; GM-CSF, granulocyte-macrophage colony-stimulating factor; HNP, human neutrophil peptide; IFN, interferon; IL, interleukin; MMP, metalloproteinase; TIMP, tissue inhibitor of MMP; TNF, tumor necrosis factor.

Unsupervised hierarchical clustering of patients with TBM by CSF mediators showed that patients with TBM-non-IRIS whose CSF cultures were positive for *M. tuberculosis* at TBM diagnosis tended to cluster at all time points with patients with TBM-IRIS (Figure 4 and Supplementary Table 7). Patients with TBM-non-IRIS who were culture positive showed mediator profiles more similar to patients with TBM-IRIS than to culture-negative patients with TBM-non-IRIS, at TBM diagnosis (Supplementary Table 8) and some of these patients also had increasing concentrations of mediators after starting ART as shown in Figure 3. Supplementary Table 9 shows the similarities between CSF findings in culture-positive patients with TBM-non-IRIS and those with TBM-IRIS and the differences between culture-positive and culture-negative patients with TBM-non-IRIS, 2 weeks after starting ART. Neutrophil counts and S100A8/A9 distinguished culture-positive patients with TBM-non-IRIS from patients with TBM-IRIS; neutrophils differentiated them at baseline (median [IQR], 3 [0–14] vs 38 [11–117] cells × 10^6^/L; *P* = .02) and 2 weeks after ART initiation (2 [0–11] vs 52 [17–244] cells × 10^6^/L; *P* = .003), and S100A8/A9 differentiated them 2 weeks after ART (15 346 [0–19 152] vs 33 500 [27 000–48 000] pg/mL; *P* = .001). Correlation analyses showed no biologically significant correlations between neutrophil and lymphocyte counts and mediator concentrations in CSF samples from patients with TBM-IRIS or TBM-non-IRIS over time (Supplementary Data).

## DISCUSSION

To our knowledge, this is the only comprehensive analysis to date of serial CSF and blood immune mediators in TBM-IRIS. Neutrophils, lymphocytes, and total protein concentrations [3], as well as all analyzed mediators (except IFN-α2), were elevated in patients with TBM-IRIS 2 weeks after ART initiation, compared with patients with TBM-non-IRIS. This widespread up-regulation of diverse mediators of diverse cellular functions suggests that both innate and adaptive immune responses are involved in TBM-IRIS pathogenesis.

Adjunctive corticosteroid treatment is associated with reduced short-term mortality in HIV-uninfected patients with TBM [15] and with symptomatic improvement in TB-IRIS [1]. In our study, increased CSF inflammation was observed after ART initiation in patients with TBM-IRIS despite corticosteroid therapy. Furthermore, a decrease of only 2 mediators was observed 2 weeks after corticosteroids were increased in dosage or restarted at TBM-IRIS presentation. Previous studies in patients with TBM also found little effect of corticosteroids on CSF cytokine or chemokine concentrations [16]. The CSF concentrations of MMP-9 were shown to decrease in patients receiving dexamethasone compared with controls early during TBM treatment, and it was postulated that this may represent a mechanism by which corticosteroids improve outcome in these patients [17]. However, in our study MMP-9 concentrations did not change after initiation of tuberculosis treatment (plus corticosteroids) in patients with either TBM-IRIS or TBM-non-IRIS, and they increased significantly after ART initiation in the TBM-IRIS group. These findings suggest that immunomodulatory treatment options more potent and specific than corticosteroids need to be explored for the prevention and/or management of TBM-IRIS.

As reported elsewhere for extrapulmonary tuberculosis, including TBM [18], pleural [19], and pericardial tuberculosis [20], a highly compartmentalized inflammatory response in CSF was seen in patients with TBM. Relatively fewer differences between TBM-IRIS and TBM-non-IRIS groups were observed for mediators in blood compared with the differences observed in CSF. Corticosteroids do modulate blood inflammatory responses in persons coinfected with tuberculosis and HIV persons [21, 22] and probably further attenuated differences between TBM-IRIS and TBM-non-IRIS groups after TBM presentation after the start of tuberculosis treatment and corticosteroids.

High baseline CSF mycobacterial load (reflected by *M. tuberculosis* culture positivity) is a risk factor for subsequent TBM-IRIS in patients with TBM [3]. This is similar to findings in cryptococcal meningitis (CM) IRIS, wherein high CSF fungal loads (reflected by quantitative culture) at CM diagnosis also predict subsequent IRIS [23]. Findings of previous studies suggested that, unlike the highly inflammatory baseline presentation being predictive of TBM-IRIS, paradoxical CM-IRIS is predicted by a paucity of inflammation at CM diagnosis, as evidenced by a lack of CSF leukocytes and/or normal protein level [23, 24] and lower CSF concentrations of cytokines and chemokines (ie, IL-6, interleukin 8, TNF, and IFN-γ) [24]. These differing findings in TBM and CM suggest that more than one pathogenic mechanism underlies neurological forms of IRIS. More recent studies in CM-IRIS have demonstrated a chemokine profile before ART in CSF that predicts subsequent IRIS risk, including higher CCL2/CXCL10 and CCL3/CXCL10 ratios [25]. Furthermore, in the same cohort of patients with CM, markers of activation and/or function expressed by natural killer cells and monocytes were compartmentalized in the CNS relative to blood before ART initiation, supporting a role for the innate immune system at the disease site in CM pathogenesis [26].

In this study, patients with TBM-non-IRIS whose CSF cultures were positive for *M. tuberculosis* at TBM diagnosis tended to have higher baseline mediator concentrations than culture-negative patients with TBM-non-IRIS and showed recurrent inflammation after ART initiation, reaching levels of magnitude comparable to those in TBM-IRIS 2 weeks after ART initiation. These increases in mediator concentrations were not seen in culture-negative patients with TBM-non-IRIS. Drug regimens in TBM are based on the same principles as pulmonary tuberculosis, rather than being informed by results of randomized controlled trials in TBM [27]. Our data support research into antimicrobial regimens containing higher-than-normal-dose rifampicin as well as a fluoroquinolone [28, 29]. Given that mycobacterial load drives inflammation during ART, improving early mycobacterial clearance from the CNS by using a more potent TBM drug regimen in HIV-infected patients with TBM may decrease the risk of TBM-IRIS.

Although initial studies explored the role of lymphocytes in TB-IRIS pathogenesis, increasing evidence implicates the innate immune system as an important contributor [9], and it is proposed that neutrophils mediate the pathology in tuberculosis [30]. Based on these results and the association we found between CSF neutrophils and TBM-IRIS [3], we investigated neutrophil-associated mediators in the CSF of our cohort. All measured neutrophil-associated mediators were increased 2 weeks after initiation of ART in the TBM-IRIS compared with the TBM-non-IRIS group. Unlike the other neutrophil-associated mediators but similar to neutrophils, S100A8/A9 was also increased in patients with TBM-IRIS compared with those with TBM-non-IRIS with baseline CSF *M. tuberculosis* culture positivity 2 weeks after ART initiation, when TBM-IRIS usually presents. Given these findings, we hypothesize that S100A8/A9 contributes to the recurrent CSF inflammation that manifests as clinical deterioration in patients with TBM-IRIS. S100A8/A9 is a protein complex that seems to have prominent immune regulatory properties, such as neutrophil chemoattraction and stimulation [31]. Several studies have shown that serum S100A8/A9 concentrations are increased in HIV-uninfected patients with pulmonary tuberculosis compared with controls, and that they are correlated with radiographic severity [32, 33]. In a murine model of tuberculosis, IL-17–induced S100A8/A9 was a key factor in neutrophil accumulation and exacerbated lung inflammation by inducing proinflammatory cytokines [33]. Given that IL-17 may therefore be important in TBM-IRIS pathogenesis, but considering the low concentrations previously found in vivo in patients with tuberculosis [20, 34], we measured IL-17A with a high-sensitivity assay. The CSF IL-17A concentrations increased significantly over time in patients with TBM-IRIS, reaching concentrations more than 8-fold that of TBM diagnosis at TBM-IRIS presentation, whereas the opposite trend occurred in patients with TBM-non-IRIS. These findings support previous suggestions that IL-17 may be important in TB-IRIS pathogenesis [34].

Although this is the first comprehensive analysis hitherto of the disease site coupled with blood immune mediators in TB-IRIS, we acknowledge certain limitations. We did not include HIV-uninfected patients with TBM as controls, and we excluded patients with neurological deterioration before ART initiation. We were therefore unable to compare the inflammatory response in TBM-IRIS to that of the “paradoxical reaction” characterized by neurological deterioration, which can occur in both HIV-infected and HIV-uninfected patients with TBM after the start of tuberculosis treatment [35]. This is a descriptive study of mediators in patients with TBM; cellular contributions to mediator production in CSF and blood were not determined by functional experiments.

Our findings have several important implications. First, oral corticosteroid therapy during TBM treatment is insufficient to prevent, or rapidly reduce, the inflammatory response that characterizes TBM-IRIS; in addition to investigations through randomized trials of different corticosteroid doses and routes of administration in the management of TBM-IRIS , alternative immunomodulatory therapies should be explored for the prevention and treatment of the disease. Second, although the adaptive immune system seems to be activated in TBM-IRIS, the sequence of immunological events in the CNS suggests a major contribution of the innate response in this condition. Cerebrospinal fluid neutrophils, which may be driven through an IL-17– and S100A8/A9-dependent pathway, are associated with the most severe CNS inflammation manifesting as TBM-IRIS; these findings are likely to direct future research into TB-IRIS immunopathogenesis and management strategies.

## Supplementary Data

Supplementary materials are available at *Clinical Infectious Diseases* online (http://cid.oxfordjournals.org). Supplementary materials consist of data provided by the author that are published to benefit the reader. The posted materials are not copyedited. The contents of all supplementary data are the sole responsibility of the authors. Questions or messages regarding errors should be addressed to the author.

Supplementary Data
