# Supplementary material for: Neutrophil-Associated Central Nervous System Inflammation in Tuberculous Meningitis Immune Reconstitution Inflammatory Syndrome
Source: Clin Infect Dis. 2014 Aug 8;59(11):1638–47. doi: 10.1093/cid/ciu641 (PMC4227574; doi:10.1093/cid/ciu641)
Supplement: Supplementary Data [file supp_59_11_1638__index.html]

Neutrophil-Associated Central Nervous System Inflammation in Tuberculous Meningitis Immune Reconstitution Inflammatory Syndrome — Neutrophil-Associated Central Nervous System Inflammation in Tuberculous Meningitis Immune Reconstitution Inflammatory Syndrome — Supplementary Data 

# Neutrophil-Associated Central Nervous System Inflammation in Tuberculous Meningitis Immune Reconstitution Inflammatory Syndrome

## Supplementary Data

Supplementary Data

**Files in this Data Supplement:**

- Supplementary Data - Doc file
