# Supplementary material for: Neutrophil-Associated Central Nervous System Inflammation in Tuberculous Meningitis Immune Reconstitution Inflammatory Syndrome
Source: Clin Infect Dis. 2014 Aug 8;59(11):1638–47. doi: 10.1093/cid/ciu641 (PMC4227574; doi:10.1093/cid/ciu641)
Supplement: Supplementary Data [file supp_ciu641_ciu641supp.doc]

**Neutrophil-associated central nervous system inflammation in tuberculous meningitis immune reconstitution inflammatory syndrome**

Suzaan Marais, Katalin A. Wilkinson, Maia Lesosky, Anna K. Coussens, Armin Deffur, Dominique J. Pepper, Charlotte Schutz, Zahiera Ismail, Graeme Meintjes, Robert J. Wilkinson

**Supplementary Data**

**Methods**

**Participants**

ART-naïve HIV-infected adults (≥ 18 years) who presented with tuberculous meningitis (TBM) were recruited from March 2009 to October 2010. Lumbar puncture (LP) was repeated within 48 hours of TBM presentation in patients who had already a diagnostic LP performed. Definite TBM was retrospectively diagnosed when *Mycobacterium tuberculosis* was cultured from cerebrospinal fluid (CSF). Probable TBM was diagnosed when a patient showed clinical, laboratory or radiological features of TBM in the absence of other infective causes for presentation including bacteria, fungi, syphilis and if suspected, viruses [1]. Paradoxical TBM-immune reconstitution inflammatory syndrome (IRIS) was diagnosed according to a published definition for TB-IRIS modified for meningitis. The definition had 3 components: (i) TBM diagnosis before starting ART and improvement on TB treatment prior to ART initiation; (ii) onset of TBM-IRIS manifestations (ie, new, recurrent, or worsening clinical features of TBM) within 3 months of ART initiation; and (iii) exclusion of alternative causes for clinical deterioration [2, 3]. As control participants, we enrolled HIV-infected ART-naïve adults (≥ 18 years) without a meningitis diagnosis who presented with symptoms and/or signs necessitating a lumbar puncture such as headache, cognitive decline or seizures. HIV infection itself is often associated with a mild to moderate CSF lymphocytic pleocytosis [4]; we therefore did not exclude patients with a mild lymphocytosis (up to 25 cells x 10^6^/L) as controls.

**Luminex multiplex assays for cytokines, chemokines, matrix metalloproteinases (MMP) and tissue inhibitors of MMP (TIMP)**

Mediators analysed in CSF and serum included: tumor necrosis factor (TNF), interferon (IFN)-γ, interleukin (IL)-2, IL-4, IL-10, IL-13, IL-1β, IL-6, IL-12p40, IL-17, IFN-α2, C-C chemokine 2 ligand (CCL2), CCL3, CCL4, C-X-C chemokine ligand 1-3 (CXCL1-3), CXCL8, granulocyte colony-stimulating factor (G-CSF) and granulocyte-macrophage (GM)-CSF. MMP-1, -2, -3, -7, -9, -10, -12 and -13, and TIMP-1 and -2 were analysed in CSF and plasma. These assays were performed in 96-well filter plates on the Bio-Plex platform (Bio-Rad Laboratories, Hercules, CA, USA) using customized Milliplex^TM^ kits (Millipore, St Charles, MO, USA).

**ELISA for cytokines, chemokines and neutrophil-associated mediators**

Mediators measured in CSF and serum samples included: CXCL10, IL-22 and IL-23 (R & D Systems, Minneapolis, USA); IL-21 (BioLegend, San Diego, USA); and IL-12p70 and IL-17A (high sensitivity assay) (eBioscience, Inc., San Diego, USA). CSF was also analysed for IL-18 (Medical and Biological Laboratories Co, Naka-ku Nagoya, Japan) and neutrophil-associated mediators: cathepsin G (DRG Diagnostics, Marburg, Germany); lipocalin-2 (RayBiotech inc. Norcross, USA); and LL-37, human neutrophil peptides (HNP) 1-3, complement (C) 5a and S100A8/A9 (Hycult Biotech inc., Uden, The Netherlands).

All assays were performed according to manufacturer’s instructions. For Luminex multiplex experiments, all CSF and blood samples from an individual patient were assayed on the same plate. Mediator concentrations were calculated with reference to a standard curve for each mediator derived from a range of concentrations of mediator standards assayed in the same manner as CSF and blood samples. Manufacturer supplied internal controls were used to validate standard curves. For ELISA experiments CSF and blood samples were assayed on separate plates. The sensitivities of the assays are presented in Supplementary Table 1.

**Results**

**Mediators not detected**

The median concentrations of IL-4, IL-12p70, IL-13, IL-21, IL-23 and MMP-12 were lower than the assay limit of detection in both blood and CSF in all subgroups (i.e. TBM-IRIS, TBM-non-IRIS and no meningitis patients) at all timepoints, whilst IL-2 (median <7 pg/ml in TBM-IRIS patients at TBM diagnosis and starting ART) and MMP-13 (median=12 pg/ml in TBM-IRIS patients at TBM diagnosis) were minimally detected in CSF of TBM-IRIS patients at the indicated timepoints. These mediators were therefore excluded from further analysis.

**Statistical analysis**

A logistic multivariate model was developed (not shown) to predict TBM-IRIS from mediators measured in CSF at time of TBM diagnosis. The resulting model was extremely unstable due to a high degree of collinearity (measured by variance inflation factor) and a small number of very high leverage individuals whereby removal resulted in instability in coefficient estimates. The variable selection process was flawed as standard methods (e.g. starting with those variables significant after a univariate test) would rest on a univariate test with low power (due to large proportion of values below the limit of detection). We therefore chose not to report the multivariate model as a supplement to univariate analyses.

**Correlation between CSF mediators and white blood cell counts**

Supplementary Table 10 shows Benjamini and Hochberg adjusted p-values [5] for correlations between neutrophil and lymphocyte counts and mediator concentrations measured in CSF in TBM-IRIS and TBM-non-IRIS patients over time. Notably, no significant correlation was observed between either cell type and any mediator in TBM-IRIS patients at any timepoint. A lack of significant correlation between mediators and cells was also observed in TBM-non-IRIS patients at TBM diagnosis. Although a statistically significant correlation between a minority of mediators and cell counts was observed at further timepoints in TBM-non-IRIS patients, these correlations were unlikely to be of biological significance, rather reflective of multiple zero values in comparator data.

**Tables**

**Supplementary Table 1. Sensitivities of Luminex multiplex and ELISA assays performed on mediators determined in cerebrospinal fluid (CSF) and blood**

| **Performed by LUMINEX multiplex *** | **CSF/Blood** | |
| --- | --- | --- |
| **G-CSF** | 3.2-16 | |
| **GM-CSF** | 3.2 | |
| **IFN-α2** | 3.2 | |
| **IL-12p40** | 3.2-16 | |
| **TNF** | 3.2 | |
| **IFN-γ** | 3.2 | |
| **IL-10** | 3.2 | |
| **IL-1β** | 3.2 | |
| **IL-6** | 3.2 | |
| **IL-17** | 3.2 | |
| **IL-2** | 3.2 | |
| **IL-4** | 3.2 | |
| **IL-13** | 3.2 | |
| **CCL2** | 3.2 | |
| **CCL3** | 3.2-16 | |
| **CCL4** | 3.2-16 | |
| **CXCL8** | 3.2 | |
| **CXCL1-3** | 3.2-16 | |
| **MMP-1** | 4.9 | |
| **MMP-2** | 12-3125 | |
| **MMP-3** | 49-195 | |
| **MMP-7** | 98 | |
| **MMP-9** | 2.4 | |
| **MMP-10** | 4.9 | |
| **MMP-12** | 391-1563 | |
| **MMP-13** | 9.8 | |
| **TIMP-1** | 19.5 | |
| **TIMP-2** | 48.8 | |
| **Performed by ELISA †** | **CSF** | **Blood** |
| **IL-12p70** | 2.5-6.5 | 5.1-6.5 |
| **IL-17A** | 0.26-0.29 | 0.28-0.35 |
| **IL-21** | 22.6-33.9 | 20.9-26.4 |
| **IL-22** | 3.8-12.3 | 6.8-7.5 |
| **IL-23** | 32-43.7 | 5.5-46.7 |
| **CXCL10** | 2.1-6.1 | 8.3-11.8 |
| **IL-18** | 4.9-7.7 | - |
| **C5a** | 549-617 | - |
| **Lipocalin-2** | 4.9-20.6 | - |
| **Cathepsin G** | 6.9-7.2 | - |
| **HNP 1-3** | 247-360 | - |
| **LL-37** | 156-255 | - |
| **S100A8/A9** | 1150-1300 | - |

**Footnotes for Supplementary Table 1:**

Units are picograms per milliliter except for Cathepsin G that is presented as units per milliliter

* For Luminex multiplex experiments, all CSF and blood samples from an individual patient were assayed on the same plate

† For ELISA experiments CSF and blood samples were assayed on separate plates

**Supplementary Table 2. Baseline cerebrospinal fluid (CSF) and blood (Bl) mediator concentrations in patients with tuberculous meningitis (TBM) and controls with no meningitis**

|  | **TBM (n=34)** | | | | | **No meningitis (n=14)** | | | | |  |  |
| --- | --- | --- | --- | --- | --- | --- | --- | --- | --- | --- | --- | --- |
| Mediator | **Blood** | | **CSF** | | Bl vs CSF | **Blood** | | **CSF** | | Bl vs CSF | Bl vs Bl | CSF vs CSF |
| pg/ml * | median | (IQR) | median | (IQR) | p-value | median | (IQR) | median | (IQR) | p-value | p-value | p-value |
| **G-CSF** | 80 | (45 -169) | 1748 | (362-6536) | <0.0001 | 42 | (21-95) | 140 | (62-231) | 0.025 | 0.084 | **< 0.0001** |
| **GM-CSF** | 0 | (0-5) | 21 | (0-69) | <0.0001 | 0 | (0-1) | 0 | (0-0) | - | - | **< 0.0001** |
| **IFN-α2** | 11 | (0-37) | 97 | (62-114) | <0.0001 | 4 | (0-11) | 58 | (47-70) | 0.0010 | 0.15 | **0.0013** |
| **IL-12p40** | 0 | (0-34) | 23 | (0-91) | 0.18 | 0 | (0-0) | 0 | (0-13) | - | - | **0.015** |
| **TNF** | 18 | (12-42) | 130 | (31-264) | <0.0001 | 16 | (9-32) | 6 | (3-13) | 0.052 | 0.38 | **< 0.0001** |
| **IFN-γ** | 8 | (0-21) | 640 | (16-1701) | <0.0001 | 0 | (0-0) | 0 | (0-0) | - | **0.0001** | **< 0.0001** |
| **IL-10** | 10 | (4-19) | 96 | (51-196) | <0.0001 | 6 | (0-16) | 0 | (0-18) | 1.00 | 0.29 | **< 0.0001** |
| **IL-1β** | 0 | (0-1) | 6 | (0-18) | 0.0005 | 0 | (0-0) | 0 | (0-0) | - | - | **0.0011** |
| **IL-6** | 7 | (4-14) | 2504 | (58-8883) | <0.0001 | 0 | (0-4) | 0 | (0-6) | - | **0.0010** | **< 0.0001** |
| **IL-22** | 0 | (0-12) | 42 | (0-160) | 0.0011 | 0 | (0-34) | 0 | (0-0) | - | - | **0.0007** |
| **IL-18** | - | - | 0 | (0-250) | - | - | - | 0 | (0-0) | - | - | - |
| **IL-17A** | 0 | (0-0) | 1 | (0-25-2.35) | < 0.0001 | 0 | (0-0.10) | 0.23 | (0-1.58) | 0.29 | - | 0.13 |
| **CXCL1-3** | 1246 | (772-2150) | 442 | (64-1558) | 0.056 | 1156 | (551-1882) | 0 | (0-48) | 0.0001 | 0.55 | **< 0.0001** |
| **CCL2** | 302 | (180-412) | 2935 | (870-7936) | <0.0001 | 402 | (308-612) | 1345 | (909-3156) | 0.0001 | **0.045** | 0.18 |
| **CCL3** | 0 | (0-0) | 82 | (49-132) | <0.0001 | 0 | (0-0) | 24 | (22-36) | 0.0011 | - | **< 0.0001** |
| **CCL4** | 16 | (0-36) | 90 | (38-151) | <0.0001 | 18 | (0-34) | 13 | (5-26) | 0.78 | 0.84 | **< 0.0001** |
| **CXCL8** | 24 | (12-44) | 1959 | (204-5353) | <0.0001 | 9 | (5-18) | 61 | (39-163) | 0.0001 | **0.0033** | **< 0.0001** |
| **CXCL10** | 1113 | (739-1620) | 6844 | (2731-8499) | <0.0001 | 730 | (553-1034) | 347 | (68-1380) | 0.30 | **0.039** | **< 0.0001** |
| **HNP 1-3** | - | - | 3578 | (803-4892) | - | - | - | 526 | (462-615) | - | - | **< 0.0001** |
| **LL-37** | - | - | 568 | (0-949) | - | - | - | 0 | (0-52) | - | - | **0.0003** |
| **Cathepsin G** | - | - | 11 | (0-15) | - | - | - | 0 | (0-8) | - | - | **0.0078** |
| **Lipocalin-2** | - | - | 1066 | (748-1169) | - | - | - | 63 | (7-124) | - | - | **< 0.0001** |
| **C5a** | - | - | 0 | (0-1320) | - | - | - | 0 | (0-0) | - | - | - |
| **S100A8/A9** | - | - | 29000 | (6000-48000) | - | - | - | 0 | (0-3250) | - | - | **0.0002** |
| **MMP-1** | 710 | (287-1681) | 142 | (23-269) | < 0.0001 | 436 | (277-626) | 2 | (0-8) | 0.0002 | 0.14 | **< 0.0001** |
| **MMP-2** | 45490 | (33600-53920) | 28790 | (19410-40760) | 0.0006 | 50140 | (46750-55670) | 13900 | (11990-17540) | 0.0002 | 0.080 | **0.0001** |
| **MMP-3** | 11290 | (7258-20430) | 1280 | (632-2040) | < 0.0001 | 5807 | (2482-10460) | 403 | (279-660) | 0.0002 | **0.023** | **< 0.0001** |
| **MMP-7** | 21260 | (14040-34860) | 314 | (205-642) | < 0.0001 | 12660 | (9526-24930) | 150 | (74-278) | 0.0002 | 0.058 | **0.0017** |
| **MMP-9** | 7914 | (5822-12550) | 25390 | (7110-69610) | 0.0004 | 10380 | (9065-13050) | 2821 | (585-6352) | 0.0034 | 0.055 | **0.0001** |
| **MMP-10** | 584 | (461-1064) | 46 | (27-75) | < 0.0001 | 892 | (522-1351) | 16 | (9-23) | 0.0002 | 0.18 | **< 0.0001** |
| **TIMP-1** | 123600 | (77620-170900) | 420100 | (188500-589900) | < 0.0001 | 109500 | (80670-123200) | 40150 | (22170-79780) | 0.0081 | 0.17 | **< 0.0001** |
| **TIMP-2** | 49800 | (40300-58040) | 48260 | (36330-68640) | 0.25 | 59820 | (43900-61980) | 33370 | (22780-39830) | 0.0046 | 0.085 | **0.0012** |

**Footnotes for Supplementary Table 2:**

*Definitions of abbreviation*s: IQR, interquartile range

**P-values for analysis between blood and CSF:**

Concentrations significantly (p<0.05) increased in CSF compared to blood

Concentrations significantly (p<0.05) decreased in CSF compared to blood

**P-values for analysis between groups:**

Significant (p<0.05) differences between groups are indicated in **bold**

* Units are picograms per milliliter except for Cathepsin G that is presented as units per milliliter

Results shown are mediator concentrations assayed with Luminex multiplex or ELISA

Statistical analyses were not performed if the medians of both comparator groups were zero

**Supplementary Table 3. Mediator concentrations in cerebrospinal fluid (CSF) and blood (Bl) of TBM-IRIS and TBM-non-IRIS patients at TBM diagnosis**

|  | **CSF** | | | |  | **Blood** | | | |  | TBM-IRIS | TBM-non-IRIS |
| --- | --- | --- | --- | --- | --- | --- | --- | --- | --- | --- | --- | --- |
| Mediator | **TBM-IRIS (n=16)** | | **TBM-non-IRIS (n=18)** | | CSF vs CSF | **TBM-IRIS (n=16)** | | **TBM-non-IRIS (n=18)** | | Bl vs Bl | Bl vs CSF | Bl vs CSF |
| pg/ml * | median | (IQR) | median | (IQR) | p-value | median | (IQR) | median | (IQR) | p-value | p-value | p-value |
| **G-CSF** | 5102 | (808-12860) | 451 | (118-2528) | **0.0035** | 88 | (73-180) | 75 | (34-169) | 0.43 | 0.0005 | 0.0011 |
| **GM-CSF** | 25 | (17-74) | 2 | (0-70) | **0.032** | 0 | (0-8) | 0 | (0-4) | - | 0.0021 | 0.0373 |
| **IFN-α2** | 112 | (81-127) | 76 | (53-104) | **0.016** | 21 | (8-54) | 4 | (0-24) | **0.030** | 0.0005 | 0.0002 |
| **IL-12p40** | 42 | (3-108) | 0 | (0-56) | 0.14 | 6 | (0-81) | 0 | (0-20) | 0.20 | 0.53 | - |
| **TNF** | 248 | (129-385) | 36 | (13-156) | **0.0002** | 20 | (13-43) | 16 | (10-38) | 0.27 | 0.0005 | 0.025 |
| **IFN-γ** | 1256 | (695-1710) | 18 | (4-970) | **0.0067** | 9 | (4-18) | 6 | (0-24) | 0.68 | 0.0005 | 0.047 |
| **IL-10** | 120 | (82-189) | 60 | (20-214) | 0.11 | 10 | (0-14) | 10 | (4-20) | 0.43 | 0.0005 | 0.0074 |
| **IL-1β** | 12 | (5-57) | 0 | (0-9) | **0.015** | 0 | (0-9) | 0 | (0-0) | - | 0.0097 | - |
| **IL-6** | 8816 | (1668-10550) | 71 | (16-3939) | **0.0003** | 10 | (5-15) | 5 | (0-10) | 0.059 | 0.0005 | 0.0005 |
| **IL-22** | 71 | (8-154) | 0 | (0-184) | 0.16 | 0 | (0-16) | 0 | (0-12) | - | 0.0032 | - |
| **IL-18** | 228 | (0-647) | 0 | (0-137) | **0.0089** | - | - | - | - | - | - | - |
| **IL17A** | 0.86 | (0.34-2.50) | 1.08 | (0-1.74) | 0.92 | 0 | (0-0) | 0 | (0-0) | - | 0.0002 | 0.0007 |
| **CXCL1-3** | 1140 | (354-2284) | 99 | (0-806) | **0.0035** | 1478 | (889-2274) | 1086 | (479-1631) | 0.18 | 0.70 | 0.0235 |
| **CCL2** | 4524 | (2031-8556) | 1160 | (699-7598) | **0.042** | 296 | (156-394) | 312 | (190-614) | 0.28 | 0.0005 | 0.0004 |
| **CCL3** | 118 | (72-190) | 56 | (38-102) | **0.0052** | 0 | (0-0) | 0 | (0-0) | - | 0.0021 | 0.0003 |
| **CCL4** | 128 | (73-264) | 56 | (22-114) | **0.010** | 20 | (0-54) | 6 | (0-18) | 0.12 | 0.0005 | 0.0004 |
| **CXCL8** | 3428 | (1854-8536) | 264 | (132-3910) | **0.0044** | 35 | (22-47) | 16 | (8-27) | **0.0049** | 0.0005 | 0.0002 |
| **CXCL10** | 7901 | (6311-8996) | 3152 | (1273-7806) | **0.0089** | 1180 | (914-2280) | 842 | (642-1476) | 0.095 | 0.0005 | 0.0008 |
| **HNP 1-3** | 4879 | (3593-5648) | 1018 | (585-3782) | **< 0.0001** | - | - | - | - | - | - | - |
| **LL-37** | 762 | (442-1117) | 428 | (0-778) | 0.064 | - | - | - | - | - | - | - |
| **Cathepsin G** | 12 | (2-18) | 8 | (0-12) | 0.10 | - | - | - | - | - | - | - |
| **Lipocalin-2** | 1114 | (1046-1192) | 845 | (314-1135) | **0.017** | - | - | - | - | - | - | - |
| **C5a** | 1044 | (172-1752) | 0 | (0-0) | **0.0053** | - | - | - | - | - | - | - |
| **S100A8/A9** | 45000 | (27500-49000) | 20500 | (0-38250) | **0.0096** | - | - | - | - | 0.49 | - | - |
| **MMP-1** | 246 | (97-634) | 28 | (8-167) | **0.0004** | 1010 | (439-3075) | 556 | (204-1004) | **0.037** | 0.0007 | 0.0004 |
| **MMP-2** | 33550 | (20570-41630) | 26310 | (18670-38790) | 0.60 | 43130 | (33530-52250) | 46170 | (35340-54770) | 0.77 | 0.016 | 0.010 |
| **MMP-3** | 1442 | (744-2006) | 844 | (613-2111) | 0.60 | 10340 | (7515-16100) | 12530 | (4328-26850) | 0.82 | 0.0006 | 0.0003 |
| **MMP-7** | 570 | (309-914) | 209 | (172-400.5) | **0.0012** | 25160 | (17120-35270) | 18260 | (12900-29180) | 0.21 | 0.0005 | 0.0002 |
| **MMP-9** | 60480 | (24540-95750) | 9368 | (1681-33420) | **0.0006** | 7258 | (5126-14450) | 8066 | (5974-10640) | 0.82 | 0.0015 | 0.49 |
| **MMP-10** | 64 | (36-85) | 39 | (23-69) | 0.10 | 600 | (381-1194) | 544 | (465-1064) | 0.97 | 0.0006 | 0.0002 |
| **TIMP-1** | 514000 | (415900-835900) | 246100 | (99360-484900) | **0.0009** | 136900 | (75910-181600) | 103200 | (86550-170800) | 0.40 | 0.0005 | 0.010 |
| **TIMP-2** | 63620 | (42400-78370) | 38280 | (34320-57320) | **0.012** | 46560 | (34590-55280) | 49800 | (41090-61620) | 0.29 | 0.019 | 0.41 |
| **MMP-9/**  **TIMP-1** | 0.1 | (0.04-0.22) | 0.04 | (0.01-0.1) | 0.51 | - | - | - | - | - | - | - |

**Footnotes for Supplementary Table 3:**

*Definitions of abbreviation*s: IQR, interquartile range

**P-values for analysis between blood and CSF:**

Concentrations significantly (p<0.05) increased in CSF compared to blood

Concentrations significantly (p<0.05) decreased in CSF compared to blood

**P-values for analysis between groups:**

Significant (p<0.05) differences between groups are indicated in **bold**

* Units are picograms per milliliter except for Cathepsin G that is presented as units per milliliter and MMP-9/TIMP-1 that is presented as a ratio Results shown are mediator concentrations assayed with Luminex multiplex or ELISA

Statistical analyses were not performed if the medians of both comparator groups were zero

**Supplementary Table 4. Mediator concentrations in cerebrospinal fluid (CSF) and blood (Bl) of TBM-IRIS and TBM-non-IRIS patients at time of starting ART**

|  | **CSF** | | | |  | **Blood** | | | |  | TBM-IRIS | TBM-non-IRIS |
| --- | --- | --- | --- | --- | --- | --- | --- | --- | --- | --- | --- | --- |
| Mediator | **TBM-IRIS (n=16)** | | **TBM-non-IRIS (n=18)** | | CSF vs CSF | **TBM-IRIS (n=16)** | | **TBM-non-IRIS (n=18)** | | Bl vs Bl | Bl vs CSF | Bl vs CSF |
| pg/ml * | median | (IQR) | median | (IQR) | p-value | median | (IQR) | median | (IQR) | p-value | p-value | p-value |
| **G-CSF** | 248 | (156-423) | 208 | (62-469) | 0.37 | 63 | (41-78) | 57 | (13-125) | 0.73 | 0.0077 | 0.019 |
| **GM-CSF** | 7 | (1-15) | 0 | (0-6) | 0.051 | 0 | (0-3) | 0 | (0-0) | - | 0.012 | - |
| **IFN-α2** | 96 | (81-132) | 66 | (54-89) | **0.0092** | 3 | (0-26) | 2 | (0-10) | 0.38 | 0.0005 | 0.0002 |
| **IL-12p40** | 100 | (41-184) | 0 | (0-58) | **0.0023** | 0 | (0-35) | 0 | (0-0) | 0.12 | 0.012 | - |
| **TNF** | 106 | (41-198) | 11 | (7-62) | **0.0023** | 18 | (12-25) | 13 | (8-31) | 0.33 | 0.0015 | 0.71 |
| **IFN-γ** | 159 | (36-403) | 4 | (0-109) | **0.0029** | 9 | (1-13) | 2 | (0-6) | 0.13 | 0.0005 | 0.064 |
| **IL-10** | 79 | (52-202) | 14 | (6-54) | **0.0009** | 12 | (8-16) | 14 | (4-40) | 0.60 | 0.0035 | 0.76 |
| **IL-1β** | 0 | (0-0) | 0 | (0-1) | - | 0 | (0-0) | 0 | (0-0) | - | - | - |
| **IL-6** | 82 | (51-386) | 14 | (2-282) | **0.043** | 0 | (0-6) | 0 | (0-0) | - | 0.0005 | 0.0026 |
| **IL-22** | 40 | (2-130) | 0 | (0-0) | **0.0013** | 5 | (0-44) | 0 | (0-39) | 0.56 | 0.10 | - |
| **IL-18** | 0 | (0-95) | 0 | (0-0) | - | - | - | - | - | - | - | - |
| **IL-17A** | 4.06 | (1.24-9.00) | 0.67 | (0-1.94) | **0.0089** | 0 | (0-0) | 0 | (0-0) | 0.19 | < 0.0001 | 0.0005 |
| **CXCL1-3** | 1177 | (487-1636) | 0 | (0-1048) | **0.0039** | 1835 | (874-2338) | 1101 | (838-2004) | 0.24 | 0.24 | 0.013 |
| **CCL2** | 2298 | (1307-3827) | 1191 | (836-1784) | **0.042** | 353 | (197-531) | 408 | (165-572) | 0.92 | 0.0005 | 0.0005 |
| **CCL3** | 56 | (46-93) | 28 | (26-53) | **0.016** | 0 | (0-0) | 0 | (0-0) | - | 0.0041 | 0.0002 |
| **CCL4** | 57 | (40-65) | 22 | (10-46) | **0.016** | 14 | (0-34) | 8 | (0-19) | 0.31 | 0.0009 | 0.020 |
| **CXCL8** | 1571 | (722-2545) | 116 | (64-1225) | **0.0028** | 20 | (12-32) | 10 | (5-19) | **0.017** | 0.0005 | 0.0002 |
| **CXCL10** | 5902 | (3005-7282) | 1163 | (640-4666) | **0.0024** | 1122 | (808-1418) | 854 | (644-1463) | 0.25 | 0.0006 | 0.11 |
| **HNP 1-3** | 1106 | (716-2305) | 531 | (427-870) | **0.0050** | - | - | - | - | - | - | - |
| **LL-37** | 0 | (0-311) | 103 | (0-342) | 0.57 | - | - | - | - | - | - | - |
| **Cathepsin G** | 0 | (0-14) | 4 | (0-11) | 0.85 | - | - | - | - | - | - | - |
| **Lipocalin-2** | 877 | (679-1013) | 242 | (125-928) | **0.020** | - | - | - | - | - | - | - |
| **C5a** | 0 | (0-952) | 0 | (0-290) | - | - | - | - | - | - | - | - |
| **S100A8/A9** | 14000 | (0-31750) | 0 | (0-28500) | 0.39 | - | - | - | - | 0.17 | - | - |
| **MMP-1** | 26 | (12-46) | 7 | (0-51) | **0.046** | 871 | (419-1322) | 488 | (282-923) | 0.12 | 0.0005 | 0.0004 |
| **MMP-2** | 34500 | (24550-51040) | 27320 | (15190-33620) | **0.030** | 61850 | (46660-79090) | 54870 | (44320-65790) | 0.19 | 0.0029 | 0.0003 |
| **MMP-3** | 829 | (365-1219) | 558 | (323-964) | 0.37 | 14530 | (8630-34650) | 11160 | (8472-25700) | 0.70 | 0.0005 | 0.0002 |
| **MMP-7** | 230 | (194-463) | 199 | (151-413) | 0.21 | 20690 | (14750-25310) | 15730 | (9946-18610) | 0.059 | 0.0005 | 0.0002 |
| **MMP-9** | 60810 | (33280-104300) | 5943 | (2606-32570) | **0.0009** | 8906 | (6400-12810) | 8574 | (7190-11780) | 0.96 | 0.0012 | 0.79 |
| **MMP-10** | 24 | (16-34) | 18 | (13-23) | 0.15 | 560 | (305-691) | 530 | (372-801) | 0.73 | 0.0005 | 0.0002 |
| **TIMP-1** | 202200 | (152400-298900) | 54890 | (42140-120800) | **< 0.0001** | 114100 | (87390-122400) | 96620 | (83560-139900) | 0.65 | 0.0010 | 0.24 |
| **TIMP-2** | 62180 | (39500-94550) | 35820 | (24340-52650) | **0.0039** | 47610 | (43690-65850) | 55550 | (47620-66060) | 0.21 | 0.066 | 0.0069 |
| **MMP-9 / TIMP-1** | 0.26 | (0.11-0.38) | 0.1 | (0.03-0.25) | 0.056 | - | - | - | - | - | - | - |

**Footnotes for Supplementary Table 4:**

*Definitions of abbreviation*s: ART, antiretroviral therapy; IQR, interquartile range

**P-values for analysis between blood and CSF:**

Concentrations significantly (p<0.05) increased in CSF compared to blood

Concentrations significantly (p<0.05) decreased in CSF compared to blood

**P-values for analysis between groups:**

Significant (p<0.05) differences between groups are indicated in **bold**

* Units are picograms per milliliter except for Cathepsin G that is presented as units per milliliter and MMP-9/TIMP-1 that is presented as a ratio

Results shown are mediator concentrations assayed with Luminex multiplex or ELISA

Statistical analyses were not performed if the medians of both comparator groups were zero

**Supplementary Table 5. Mediator concentrations in cerebrospinal fluid (CSF) and blood (Bl) of TBM-IRIS and TBM-non-IRIS patients at TBM-IRIS or 2 weeks after ART initiation (TBM-non-IRIS)**

|  | **CSF** | | | |  | **Blood** | | | |  | TBM-IRIS | TBM-  non-IRIS |
| --- | --- | --- | --- | --- | --- | --- | --- | --- | --- | --- | --- | --- |
| Mediator | **TBM-IRIS (n=16)** | | **TBM-non-IRIS (n=18)** | | CSF vs CSF | **TBM-IRIS**  **(n=16)** | | **TBM-non-IRIS (n=18)** | | Bl vs Bl | Bl vs CSF | Bl vs CSF |
| pg/ml * | median | (IQR) | median | (IQR) | p-value | median | (IQR) | median | (IQR) | p-value | p-value | p-value |
| **G-CSF** | 1572 | (622-2554) | 196 | (57-640) | **0.0008** | 64 | (55-108) | 49 | (25-106) | 0.15 | 0.0005 | 0.0020 |
| **GM-CSF** | 12 | (5-22) | 0 | (0-16) | **0.019** | 0 | (0-7) | 0 | (0-0) | - | 0.020 | - |
| **IFN-α2** | 94 | (63-128) | 66 | (50-100) | 0.062 | 9 | (0-21) | 4 | (0-11) | 0.18 | 0.0005 | 0.0002 |
| **IL-12p40** | 168 | (78-499) | 0 | (0-34) | **0.0003** | 9 | (0-50) | 0 | (0-2) | 0.066 | 0.0041 | - |
| **TNF** | 264 | (145-475) | 6 | (0-63) | **< 0.0001** | 22 | (15-40) | 14 | (6-26) | **0.045** | 0.0005 | 0.85 |
| **IFN-γ** | 376 | (268-788) | 0 | (0-105) | **< 0.0001** | 12 | (6-22) | 0 | (0-13) | **0.030** | 0.0005 | - |
| **IL-10** | 304 | (162-576) | 6 | (0-116) | **0.0002** | 11 | (6-19) | 16 | (4-42) | 0.33 | 0.0005 | 0.93 |
| **IL-1β** | 3 | (0-13) | 0 | (0-0) | **0.029** | 0 | (0-3) | 0 | (0-0) | - | 0.032 | - |
| **IL-6** | 772 | (366-5152) | 10 | (0-500) | **0.0006** | 6 | (0-13) | 0 | (0-7) | 0.23 | 0.0005 | 0.034 |
| **IL-22** | 162 | (25-875) | 0 | (0-0) | **0.0001** | 6 | (0-39) | 0 | (0-22) | 0.52 | 0.0001 | - |
| **IL-18** | 123 | (0-2030) | 0 | (0-0) | **0.0008** | - | - | - | - | - | - | - |
| **IL-17A** | 7.6 | (0.81-14.9) | 0.36 | (0-4.07) | **0.0017** | 0 | (0-0) | 0 | (0-0) | 0.54 | 0.0005 | 0.0068 |
| **CXCL1-3** | 1435 | (850-2242) | 0 | (0-1079) | **0.0011** | 1966 | (987-2605) | 1150 | (897-1852) | 0.095 | 0.39 | 0.012 |
| **CCL2** | 2792 | (1798-7032) | 1005 | (606-2274) | **0.0039** | 296 | (211-526) | 402 | (253-552) | 0.32 | 0.0005 | 0.0006 |
| **CCL3** | 84 | (68-124) | 32 | (28-56) | **0.0008** | 0 | (0-0) | 0 | (0-0) | - | 0.0009 | 0.0002 |
| **CCL4** | 108 | (67-155) | 20 | (14-46) | **< 0.0001** | 36 | (16-55) | 15 | (3-25) | **0.047** | 0.0005 | 0.099 |
| **CXCL8** | 3461 | (2075-7132) | 91 | (43-1839) | **0.0001** | 34 | (15-58) | 8 | (2-23) | **0.0041** | 0.0005 | 0.0002 |
| **CXCL10** | 6786 | (5734-8379) | 233 | (28-4605) | **0.0001** | 1175 | (744-1694) | 406 | (227-859) | **0.0014** | 0.0005 | 0.26 |
| **HNP 1-3** | 4406 | (2867-6228) | 633 | (417-1568) | **< 0.0001** | - | - | - | - | - | - | - |
| **LL-37** | 719 | (314-1323) | 252 | (0-353) | **0.0071** | - | - | - | - | - | - | - |
| **Cathepsin G** | 11 | (9-14) | 0 | (0-10) | **0.0068** | - | - | - | - | - | - | - |
| **Lipocalin-2** | 1150 | (1050-1280) | 274 | (21-943) | **0.0012** | - | - | - | - | - | - | - |
| **C5a** | 1050 | (0-10350) | 0 | (0-204) | **0.015** | - | - | - | - | - | - | - |
| **S100A8/A9** | 33500 | (27000-48000) | 0 | (0-17500) | **< 0.0001** | - | - | - | - | 0.46 | - | - |
| **MMP-1** | 73 | (34-316) | 6 | (0-52) | **0.0013** | 844 | (466-1318) | 486 | (286-665) | 0.060 | 0.0012 | 0.0004 |
| **MMP-2** | 49010 | (32970-70180) | 20800 | (13480-35820) | **0.0004** | 51490 | (46720-64140) | 51440 | (42300-64190) | 0.70 | 0.55 | 0.0041 |
| **MMP-3** | 1453 | (461-2846) | 632 | (371-1006) | **0.036** | 16970 | (6158-33490) | 22420 | (8006-110400) | 0.31 | < 0.0001 | 0.0003 |
| **MMP-7** | 426 | (282-816) | 183 | (120-414) | **0.0058** | 18020 | (16020-39510) | 13460 | (10560-17510) | **0.022** | 0.0005 | 0.0002 |
| **MMP-9** | 139000 | (84410-180400) | 3828 | (772-28150) | **0.0001** | 12400 | (9281-18330) | 9232 | (7250-14260) | 0.18 | 0.0006 | 0.90 |
| **MMP-10** | 40 | (26-93) | 16 | (14-25) | **0.0039** | 563 | (312-751) | 533 | (284-992) | 0.89 | 0.0005 | 0.0002 |
| **TIMP-1** | 453100 | (149700-952100) | 57380 | (33390-110200) | **< 0.0001** | 114200 | (72400-170700) | 90760 | (71450-171800) | 0.75 | 0.0021 | 0.15 |
| **TIMP-2** | 68380 | (52210-91680) | 42990 | (24410-52720) | **0.0024** | 41280 | (38990-59190) | 58130 | (54690-63580) | **0.046** | 0.0035 | 0.041 |
| **MMP-9/ TIMP-1** | 0.34 | (0.09-1.05) | 0.04 | (0.01-0.51) | 0.029 | - | - | - | - | - | - | - |

**Footnotes for Supplementary Table 5:**

*Definitions of abbreviation*s: ART, antiretroviral therapy; IQR, interquartile range

**P-values for analysis between blood and CSF:**

Concentrations significantly (p<0.05) increased in CSF compared to blood

Concentrations significantly (p<0.05) decreased in CSF compared to blood

**P-values for analysis between groups:**

Significant (p<0.05) differences between groups are indicated in **bold**

* Units are picograms per milliliter except for Cathepsin G that is presented as units per milliliter and MMP-9/TIMP-1 that is presented as a ratio

Results shown are mediator concentrations assayed with Luminex multiplex or ELISA

Statistical analyses were not performed if the medians of both comparator groups were zero

**Supplementary Table 6. Comparison of cerebrospinal fluid (CSF) mediator concentrations between timepoints in TBM patients who A) developed TBM- IRIS and B) those who did not (TBM-non-IRIS)**

| **A) TBM-IRIS (n=16)** | | | | | | | | | | | |
| --- | --- | --- | --- | --- | --- | --- | --- | --- | --- | --- | --- |
| Mediator | **1: TBM diagnosis** | | **2: Starting ART** | | **3: TBM-IRIS diagnosis** | | **4: 2 weeks after TBM-IRIS** | | 2 vs 1 | 3 vs 2 | 3 vs 1 |
| pg/ml * | median | (IQR) | median | (IQR) | median | (IQR) | median | (IQR) | p-value | p-value | p-value |
| **G-CSF** | 5102 | (808-12860) | 248 | (156-423) | 1572 | (622-2554) | 666 | (168-941) | 0.0007 | 0.0005 | 0.01 |
| **GM-CSF** | 25 | (17-74) | 7 | (1-15) | 12 | (5-22) | 5 | (0-20) | 0.0008 | 0.19 | 0.0021 |
| **IFN-α2** | 112 | (81-127) | 96 | (81-132) | 94 | (63-128) | 131 | (97-153) | 0.45 | 1.00 | 0.55 |
| **IL-12p40** | 42 | (3-108) | 100 | (41-184) | 168 | (78-499) | 156 | (62-298) | 0.012 | 0.012 | 0.0004 |
| **TNF** | 248 | (129-385) | 106 | (41-198) | 264 | (145-475) | 206 | (157-367) | 0.0035 | 0.0029 | 0.21 |
| **IFN-γ** | 1256 | (695-1710) | 159 | (36-403) | 376 | (268-788) | 286 | (80-577) | 0.0009 | 0.0077 | 0.019 |
| **IL-10** | 120 | (82-189) | 79 | (52-202) | 304 | (162-576) | 335 | (130-580) | 0.020 | 0.0005 | 0.001 |
| **IL-1β** | 12 | (5-57) | 0 | (0-0) | 3 | (0-13) | 0 | (0-12) | 0.0017 | 0.022 | 0.0017 |
| **IL-6** | 8816 | (1668-10550) | 82 | (51-386) | 772 | (366-5152) | 372 | (114-617) | 0.0005 | 0.0005 | 0.0025 |
| **IL-22** | 71 | (8-154) | 40 | (2-130) | 162 | (25-875) | 132 | (16-498) | 0.46 | 0.0067 | 0.022 |
| **IL-18** | 228 | (0-647) | 0 | (0-95) | 123 | (0-2030) | 75 | (0-449) | 0.0049 | 0.0068 | 0.59 |
| **IL-17A** | 0.86 | (0.34-2.50) | 4.06 | (1.24-9.00) | 7.6 | (0.81-14.9) | 3.75 | (0.52-12.30) | 0.010 | 0.25 | 0.0066 |
| **CXCL1-3** | 1140 | (354-2284) | 1177 | (487-1636) | 1435 | (850-2242) | 1756 | (1079-2943) | 0.39 | 0.14 | 0.86 |
| **CCL2** | 4524 | (2031-8556) | 2298 | (1307-3827) | 2792 | (1798-7032) | 2540 | (2058-5832) | 0.025 | 0.011 | 0.19 |
| **CCL3** | 118 | (72-190) | 56 | (46-93) | 84 | (68-124) | 95 | (50-128) | 0.0021 | 0.028 | 0.17 |
| **CCL4** | 128 | (73-264) | 57 | (40-65) | 108 | (67-155) | 90 | (61-118) | 0.0012 | 0.0009 | 0.13 |
| **CXCL8** | 3428 | (1854-8536) | 1571 | (722-2545) | 3461 | (2075-7132) | 3491 | (1216-6316) | 0.021 | 0.0009 | 0.94 |
| **CXCL10** | 7901 | (6311-8996) | 5902 | (3005-7282) | 6786 | (5734-8379) | 6668 | (4395-7761) | 0.0048 | 0.0066 | 0.22 |
| **HNP 1-3** | 4879 | (3593-5648) | 1106 | (716-2305) | 4406 | (2867-6228) | 1948 | (1254-3350) | 0.0009 | 0.0029 | 0.29 |
| **LL-37** | 762 | (442-1117) | 0 | (0-311) | 719 | (314-1323) | 0 | (0-511) | 0.0005 | 0.0007 | 0.97 |
| **Cathepsin G** | 12 | (2-18) | 0 | (0-14) | 11 | (9-14) | 10 | (0-24) | 0.07 | 0.023 | 0.92 |
| **Lipocalin-2** | 1114 | (1046-1192) | 877 | (679-1013) | 1150 | (1050-1280) | 1191 | (889-1295) | 0.0005 | 0.0007 | 0.39 |
| **C5a** | 1044 | (172-1752) | 0 | (0-952) | 1050 | (0-10350) | 1043 | (434-2351) | 0.0076 | 0.0039 | 0.28 |
| **S100A8/A9** | 45000 | (27500-49000) | 14000 | (0-31750) | 33500 | (27000-48000) | 25500 | (17500-38750) | 0.0015 | 0.011 | 0.90 |
| **MMP-1** | 246 | (97-634) | 26 | (12-46) | 73 | (34-316) | 32 | (22-89) | < 0.0001 | 0.0009 | 0.21 |
| **MMP-2** | 33550 | (20570-41630) | 34500 | (24550-51040) | 49010 | (32970-70180) | 47240 | (28779-83534) | 0.17 | 0.011 | 0.0007 |
| **MMP-3** | 1442 | (744-2006) | 829 | (365-1219) | 1453 | (461-2846) | 1273 | (544-1942) | 0.0041 | 0.0029 | 0.62 |
| **MMP-7** | 570 | (309-914) | 230 | (194-463) | 426 | (282-816) | 396 | (282-613) | 0.012 | 0.041 | 0.72 |
| **MMP-9** | 60480 | (24540-95750) | 60810 | (33280-104300) | 139000 | (84410-180400) | 165161 | (136793-206467) | 0.78 | 0.0077 | 0.0012 |
| **MMP-10** | 64 | (36-85) | 24 | (16-34) | 40 | (26-93) | 50 | (30-83) | 0.0008 | 0.0024 | 0.16 |
| **TIMP-1** | 514000 | (415900-835900) | 202200 | (152400-298900) | 453100 | (149700-952100) | 289227 | (173023-493628) | 0.0009 | 0.012 | 0.11 |
| **TIMP-2** | 63620 | (42400-78370) | 62180 | (39500-94550) | 68380 | (52210-91680) | 70979 | (58889-86093) | 0.59 | 0.59 | 0.78 |
| **MMP-9/ TIMP-1** | 0.1 | (0.04-0.22) | 0.26 | (0.11-0.38) | 0.34 | (0.09-1.05) | 0.55 | (0.20-0.91) | 0.009 | 0.053 | 0.0029 |

**Footnotes for Supplementary Table 6A:**

*Definitions of abbreviation*s: ART, antiretroviral therapy; IQR, interquartile range

**P-values for analysis between blood and CSF:**

Concentrations significantly (p<0.05) increased at later compared to earlier timepoint

Concentrations significantly (p<0.05) decreased at later compared to earlier timepoint

* Units are picograms per milliliter except for Cathepsin G that is presented as units per milliliter and MMP-9/TIMP-1 that is presented as a ratio

Results shown are mediator concentrations assayed with Luminex multiplex or ELISA

Statistical analyses were not performed if the medians of both comparator groups were zero

| **B) TBM-non-IRIS (n=18)** | | | | | | | | |  |
| --- | --- | --- | --- | --- | --- | --- | --- | --- | --- |
| Mediator | **1: TBM diagnosis** | | **2: Starting ART** | | **3: 2 weeks after starting ART** | | 2 vs 1 | 3 vs 2 | 3 vs 1 |
| pg/ml * | median | (IQR) | median | (IQR) | median | (IQR) | p-value | p-value | p-value |
| **G-CSF** | 451 | (118-2528) | 208 | (62-469) | 196 | (57-640) | 0.0020 | 0.79 | 0.013 |
| **GM-CSF** | 2 | (0-70) | 0 | (0-6) | 0 | (0-16) | 0.013 | - | 0.020 |
| **IFN-α2** | 76 | (53-104) | 66 | (54-89) | 66 | (50-100) | 0.27 | 0.88 | 0.56 |
| **IL-12p40** | 0 | (0-56) | 0 | (0-58) | 0 | (0-34) | - | - | - |
| **TNF** | 36 | (13-156) | 11 | (7-62) | 6 | (0-63) | 0.0090 | 0.51 | 0.0025 |
| **IFN-γ** | 18 | (4-970) | 4 | (0-109) | 0 | (0-105) | 0.0061 | 0.41 | 0.0041 |
| **IL-10** | 60 | (20-214) | 14 | (6-54) | 6 | (0-116) | 0.0031 | 0.55 | 0.058 |
| **IL-1β** | 0 | (0-9) | 0 | (0-1) | 0 | (0-0) | - | - | - |
| **IL-6** | 71 | (16-3939) | 14 | (2-282) | 10 | (0-500) | 0.0014 | 0.81 | 0.0015 |
| **IL-22** | 0 | (0-184) | 0 | (0-0) | 0 | (0-0) | - | - | - |
| **IL-18** | 0 | (0-137) | 0 | (0-0) | 0 | (0-0) | - | - | - |
| **IL-17A** | 1.08 | (0-1.74) | 0.67 | (0-1.94) | 0.36 | (0-4.07) | 0.64 | 0.74 | 0.65 |
| **CXCL1-3** | 99 | (0-806) | 0 | (0-1048) | 0 | (0-1079) | 0.84 | - | 0.57 |
| **CCL2** | 1160 | (699-7598) | 1191 | (836-1784) | 1005 | (606-2274) | 0.15 | 0.91 | 0.074 |
| **CCL3** | 56 | (38-102) | 28 | (26-53) | 32 | (28-56) | 0.013 | 0.90 | 0.012 |
| **CCL4** | 56 | (22-114) | 22 | (10-46) | 20 | (14-46) | 0.0035 | 0.51 | 0.0005 |
| **CXCL8** | 264 | (132-3910) | 116 | (64-1225) | 91 | (43-1839) | 0.0079 | 0.83 | 0.0069 |
| **CXCL10** | 3152 | (1273-7806) | 1163 | (640-4666) | 233 | (28-4605) | 0.0027 | 0.16 | 0.0004 |
| **HNP 1-3** | 1018 | (585-3782) | 531 | (427-870) | 633 | (417-1568) | 0.0027 | 0.97 | 0.15 |
| **LL-37** | 428 | (0-778) | 103 | (0-342) | 252 | (0-353) | 0.0081 | 0.29 | 0.14 |
| **Cathepsin G** | 8 | (0-12) | 4 | (0-11) | 0 | (0-10) | 0.53 | 0.96 | 0.082 |
| **Lipocalin-2** | 845 | (314-1135) | 242 | (125-928) | 274 | (21-943) | 0.0035 | 0.96 | 0.012 |
| **C5a** | 0 | (0-0) | 0 | (0-290) | 0 | (0-204) | - | - | - |
| **S100A8/A9** | 20500 | (0-38250) | 0 | (0-28500) | 0 | (0-17500) | 0.83 | - | 0.043 |
| **MMP-1** | 28 | (8-167) | 7 | (0-51) | 6 | (0-52) | 0.17 | 0.70 | 0.16 |
| **MMP-2** | 26310 | (18670-38790) | 27320 | (15190-33620) | 20800 | (13480-35820) | 0.24 | 0.60 | 0.24 |
| **MMP-3** | 844 | (613-2111) | 558 | (323-964) | 632 | (371-1006) | 0.029 | 0.22 | 0.14 |
| **MMP-7** | 209 | (172-400) | 199 | (151-413) | 183 | (120-414) | 0.34 | 0.24 | 0.16 |
| **MMP-9** | 9368 | (1681-33420) | 5943 | (2606-32570) | 3828 | (772-28150) | 0.76 | 0.89 | 0.90 |
| **MMP-10** | 39 | (23-69) | 18 | (13-23) | 16 | (14-25) | 0.0008 | 0.49 | 0.0003 |
| **TIMP-1** | 246100 | (99360-484900) | 54890 | (42140-120800) | 57380 | (33390-110200) | 0.0015 | 0.28 | 0.010 |
| **TIMP-2** | 38280 | (34320-57320) | 35820 | (24340-52650) | 42990 | (24410-52720) | 0.074 | 0.54 | 0.54 |
| **MMP-9/ TIMP-1** | 0.04 | (0.01-0.10) | 0.1 | (0.03-0.25) | 0.04 | (0.01-0.51) | 0.012 | 1.00 | 0.067 |

**Footnotes for Supplementary Table 6B:**

*Definitions of abbreviation*s: ART, antiretroviral therapy; IQR, interquartile range

**P-values for analysis between blood and CSF:**

Concentrations significantly (p<0.05) increased at later compared to earlier timepoint

Concentrations significantly (p<0.05) decreased at later compared to earlier timepoint

* Units are picograms per milliliter except for Cathepsin G that is presented as units per milliliter and MMP-9/TIMP-1 that is presented as a ratio

Results shown are mediator concentrations assayed with Luminex multiplex or ELISA

Statistical analyses were not performed if the medians of both comparator groups were zero

**Supplementary Table 7. Jaccard similarity indices for testing cluster-wise stability of hierarchical clustering analyses of tuberculous meningitis (TBM) patients (n=34) over time***

| Number of clusters | | 2 | | 3 | | 4 | |
| --- | --- | --- | --- | --- | --- | --- | --- |
| Timepoint | Resampling method |  |  |  |  |  |  |
| **TBM diagnosis** |  |  |  |  |  |  |  |
|  | Subset | 0.8 | -0.053 | 0.73 | -0.026 | 0.79 | -0.037 |
|  | Bootstrap | 0.82 | -0.065 | 0.79 | -0.015 | 0.84 | -0.048 |
| **ART start** |  |  |  |  |  |  |  |
|  | Subset | 0.75 | -0.16 | 0.81 | -0.047 | 0.77 | -0.18 |
|  | Bootstrap | 0.77 | -0.14 | 0.88 | -0.038 | 0.78 | -0.22 |
| **2 weeks post ART †** |  |  |  |  |  |  |  |
|  | Subset | 0.71 | -0.1 | 0.79 | -0.091 | 0.71 | -0.14 |
|  | Bootstrap | 0.73 | -0.14 | 0.83 | -0.12 | 0.76 | -0.15 |

**Footnotes for Supplementary Table 7:**

*Definitions of abbreviations*: ART, antiretroviral therapy

* Mean Jaccard index values with standard deviations of clustering analysis (presented in Figure 4) are shown [6]

† For patients who developed TBM-IRIS, the 2 weeks post ART timepoint indicates findings at time of TBM-IRIS

**Interpretation of the Jaccard index:**

A valid stable cluster should yield a mean Jaccard similarity value of ≥0.75

Highly stable clusters will be >0.85, and values between 0.6 and 0.75 should be taken as indicative of patterns in the data but exactly which points belong to which clusters is doubtful

**Supplementary Table 8. Comparison of cerebrospinal fluid (CSF) mediator concentrations between TBM-IRIS, CSF *M. tuberculosis* culture-positive TBM-non-IRIS (Cult pos NI) and CSF *M. tuberculosis* culture-negative TBM-non-IRIS (Cult neg NI) patients at TBM diagnosis**

| **TBM diagnosis** | | | | | | | | |
| --- | --- | --- | --- | --- | --- | --- | --- | --- |
| Mediator | **TBM-IRIS (n=16)** | | **Cult pos TBM- non-IRIS (n=6)** | | **Cult neg TBM- non-IRIS (n=12)** | | Cult pos NI vs TBM-IRIS | Cult pos NI vs Cult neg NI |
| pg/ml * | median | (IQR) | median | (IQR) | median | (IQR) | p-value | p-value |
| **Neutrophls** | 38 | (11-117) | 3 | (0-14) | 10 | (0-43) | 0.020 | 0.37 |
| **Lymphocytes** | 216 | (120-419) | 120 | (20-214) | 94 | (54-440) | 0.083 | 0.89 |
| **G-CSF** | 5102 | (808-12860) | 4392 | (1573-9708) | 260 | (84-1203) | 0.74 | 0.0043 |
| **GM-CSF** | 25 | (17-74) | 76 | (21-159) | 0 | (0-3) | 0.20 | 0.0016 |
| **IFN-α2** | 112 | (81-127) | 74 | (52-110) | 82 | (54-102) | 0.083 | 0.96 |
| **IL-12p40** | 42 | (3-108) | 67 | (0-106) | 0 | (0-26) | 0.91 | 0.11 |
| **TNF** | 248 | (129-385) | 178 | (84-257) | 29 | (10-42) | 0.20 | 0.028 |
| **IFN-γ** | 1256 | (695-1710) | 1337 | (389-2647) | 12 | (1-20) | 0.91 | 0.027 |
| **IL-10** | 120 | (82-189) | 175 | (76-332) | 48 | (14-102) | 0.48 | 0.11 |
| **IL-1β** | 12 | (5-57) | 14 | (9-58) | 0 | (0-0) | 0.58 | 0.0002 |
| **IL-6** | 8816 | (1668-10550) | 2931 | (770-7926) | 45 | (12-1511) | 0.097 | 0.035 |
| **IL-22** | 71 | (8-154) | 19 | (0-295) | 0 | (0-147) | 0.63 | 0.49 |
| **IL-18** | 228 | (0-647) | 64 | (0-172) | 0 | (0-0) | 0.15 | 0.31 |
| **IL-17A** | 0.86 | (0.34-2.50) | 1.09 | (0-1.44) | 1.06 | (0.14-2.70) | 0.58 | 0.60 |
| **CXCL1-3** | 1140 | (354-2284) | 1334 | (477-2084) | 32 | (0-134) | 0.97 | 0.013 |
| **CCL2** | 4524 | (2031-8556) | 7676 | (5461-8483) | 765 | (550-1252) | 0.29 | 0.0043 |
| **CCL3** | 118 | (72-190) | 96 | (54-125) | 43 | (28-71) | 0.29 | 0.068 |
| **CCL4** | 128 | (73-264) | 104 | (68-127) | 37 | (20-61) | 0.36 | 0.17 |
| **CXCL8** | 3428 | (1854-8536) | 5350 | (2606-6349) | 192 | (118-417) | 0.80 | 0.011 |
| **CXCL10** | 7901 | (6311-8996) | 7978 | (5803-8779) | 2653 | (1248-3479) | 0.85 | 0.068 |
| **HNP 1-3** | 4879 | (3593-5648) | 2858 | (1590-4688) | 642 | (566-1301) | 0.030 | 0.083 |
| **LL-37** | 762 | (442-1117) | 597 | (0-1174) | 400 | (0-711) | 0.48 | 0.36 |
| **Cathepsin G** | 12 | (2-18) | 6 | (0-14) | 8 | (0-13) | 0.33 | 0.92 |
| **Lipocalin-2** | 1114 | (1046-1192) | 1183 | (765-1317) | 632 | (281-998) | 0.85 | 0.12 |
| **C5a** | 1044 | (172-1752) | 793 | (0-2435) | 0 | (0-0) | 0.91 | 0.012 |
| **S100A8/A9** | 45000 | (27500-49000) | 30029 | (18231-43575) | 4123 | (0-34505) | 0.20 | 0.23 |
| **MMP-1** | 246 | (97-634) | 48 | (0-216) | 28 | (10-149) | 0.025 | 0.96 |
| **MMP-2** | 33550 | (20570-41630) | 25017 | (17394-29308) | 31652 | (18021-50055) | 0.36 | 0.37 |
| **MMP-3** | 1442 | (744-2006) | 844 | (611-1907) | 830 | (585-2192) | 0.44 | 0.89 |
| **MMP-7** | 570 | (309-914) | 192 | (174-239) | 216 | (164-648) | 0.0010 | 0.43 |
| **MMP-9** | 60480 | (24540-95750) | 54726 | (20949-87491) | 2881 | (1323-12475) | 0.80 | 0.0057 |
| **MMP-10** | 64 | (36-85) | 32 | (21-67) | 40 | (26-70) | 0.10 | 0.35 |
| **TIMP-1** | 514000 | (415900-835900) | 280424 | (145754-543403) | 242091 | (92086-472705) | 0.043 | 0.74 |
| **TIMP-2** | 63620 | (42400-78370) | 47942 | (36330-71838) | 36974 | (31167-48888) | 0.25 | 0.21 |
| **MMP-9/**  **TIMP-1** | 0.1 | (0.04-0.22) | 0.13 | (0.07-0.36) | 0.02 | (0-0.04) | 0.29 | 0.0032 |

**Footnotes for Supplementary Table 8:**

*Definitions of abbreviations*: IQR, interquartile range

**P-values for analysis between groups**

Concentrations significantly (p<0.05) increased in culture positive TBM-non-IRIS patients compared to comparator group

Concentrations significantly (p<0.05) decreased in culture positive TBM-non-IRIS patients compared to comparator group

* Units are picograms per milliliter except for Cathepsin G that is presented as units per milliliter and MMP-9/TIMP-1 that is presented as a ratio

Results shown are mediator concentrations assayed with Luminex multiplex or ELISA

**Supplementary Table 9. Comparison of cerebrospinal fluid (CSF) mediator concentrations between TBM-IRIS, CSF *M. tuberculosis* culture positive TBM-non-IRIS (Cult pos NI) and CSF *M. tuberculosis* culture negative TBM-non-IRIS (Cult neg NI) patients at TBM-IRIS or 2 weeks after starting ART (TBM-non-IRIS)**

| **TBM-IRIS/ 2 weeks after starting ART (TBM-non-IRIS)** | | | | | | | | |
| --- | --- | --- | --- | --- | --- | --- | --- | --- |
| Mediator | **TBM-IRIS (n=16)** | | **Cult pos TBM-non-IRIS (n=6)** | | **Cult neg TBM-non-IRIS (n=12)** | | Cult pos NI vs TBM-IRIS | Cult pos NI vs Cult neg NI |
| pg/ml * | median | (IQR) | median | (IQR) | median | (IQR) | p-value | p-value |
| **Neutrophils** | 52 | (17-244) | 2 | (0-11) | 0 | (0-0) | 0.0027 | 0.045 |
| **Lymphocytes** | 208 | (90-363) | 54 | (6-142) | 22 | (5-40) | 0.011 | 0.22 |
| **G-CSF** | 1572 | (622-2554) | 645 | (408-1729) | 74 | (51-231) | 0.17 | 0.0076 |
| **GM-CSF** | 12 | (5-22) | 19 | (4-28) | 0 | (0-0) | 0.77 | 0.0056 |
| **IFN-α2** | 94 | (63-128) | 98 | (53-142) | 58 | (37-86) | 0.80 | 0.15 |
| **IL-12p40** | 168 | (78-499) | 34 | (0-221) | 0 | (0-0) | 0.11 | 0.015 |
| **TNF** | 264 | (145-475) | 126 | (28-214) | 0 | (0-14) | 0.043 | 0.0038 |
| **IFN-γ** | 376 | (268-788) | 274 | (58-413) | 0 | (0-2) | 0.17 | 0.0040 |
| **IL-10** | 304 | (162-576) | 211 | (45-374) | 0 | (0-14) | 0.29 | 0.0020 |
| **IL-1β** | 3 | (0-13) | 2 | (0-9) | 0 | (0-0) | 0.72 | 0.012 |
| **IL-6** | 772 | (366-5152) | 522 | (122-2183) | 3 | (0-19) | 0.40 | 0.030 |
| **IL-22** | 162 | (25-875) | 56 | (0-233) | 0 | (0-0) | 0.15 | 0.012 |
| **IL-18** | 123 | (0-2030) | 0 | (0-331) | 0 | (0-0) | 0.11 | - |
| **IL-17A** | 7.6 | (0.81-14.9) | 2.54 | (0.25-8.81) | 0.14 | (0-0.96) | 0.20 | 0.16 |
| **CXCL1-3** | 1435 | (850-2242) | 1137 | (643-2316) | 0 | (0-62) | 0.69 | 0.010 |
| **CCL2** | 2792 | (1798-7032) | 2332 | (1824-5187) | 684 | (534-1508) | 0.80 | 0.0076 |
| **CCL3** | 84 | (68-124) | 52 | (38-102) | 30 | (26-35) | 0.13 | 0.024 |
| **CCL4** | 108 | (67-155) | 50 | (30-102) | 16 | (2-32) | 0.020 | 0.011 |
| **CXCL8** | 3461 | (2075-7132) | 2753 | (1359-4138) | 57 | (27-102) | 0.29 | 0.0027 |
| **CXCL10** | 6786 | (5734-8379) | 5548 | (3370-7996) | 70 | (0-516) | 0.25 | 0.0022 |
| **HNP 1-3** | 4406 | (2867-6228) | 2443 | (919-6219) | 480 | (410-658) | 0.22 | 0.035 |
| **LL-37** | 719 | (314-1323) | 384 | (0-1358) | 206 | (0-331) | 0.32 | 0.21 |
| **Cathepsin G** | 11 | (9-14) | 4 | (0-25) | 0 | (0-10) | 0.15 | 0.64 |
| **Lipocalin-2** | 1150 | (1050-1280) | 1177 | (747-1350) | 140 | (0-395) | 0.91 | 0.0022 |
| **C5a** | 1050 | (0-10350) | 408 | (0-6801) | 0 | (0-0) | 0.45 | 0.072 |
| **S100A8/A9** | 33500 | (27000-48000) | 15346 | (0-19152) | 0 | (0-10120) | 0.0010 | 0.20 |
| **MMP-1** | 73 | (34-316) | 101 | (8-337) | 2 | (0-11) | 0.74 | 0.030 |
| **MMP-2** | 49010 | (32970-70180) | 28930 | (14318-44514) | 18826 | (12295-31445) | 0.071 | 0.43 |
| **MMP-3** | 1453 | (461-2846) | 856 | (564-2273) | 458 | (297-909) | 0.53 | 0.15 |
| **MMP-7** | 426 | (282-816) | 288 | (150-438) | 155 | (100-383) | 0.18 | 0.28 |
| **MMP-9** | 139000 | (84410-180400) | 87968 | (19304-268088) | 938 | (302-9996) | 0.48 | 0.0043 |
| **MMP-10** | 40 | (26-93) | 16 | (11-27) | 16 | (14-25) | 0.039 | 0.71 |
| **TIMP-1** | 453100 | (149700-952100) | 91331 | (41885-277476) | 45729 | (26602-91415) | 0.017 | 0.15 |
| **TIMP-2** | 68380 | (52210-91680) | 29885 | (23981-53582) | 46000 | (24319-55934) | 0.014 | 0.61 |
| **MMP-9/ TIMP-1** | 0.34 | (0.09-1.05) | 1.28 | (0.04-2.43) | 0.03 | (0.01-0.09) | 0.440 | 0.017 |

**Footnotes for Supplementary Table 9:**

*Definitions of abbreviations*: ART, antiretroviral therapy; IQR, interquartile range

**P-values for analysis between groups**

Concentrations significantly (p<0.05) increased in culture positive TBM-non-IRIS patients compared to comparator group

Concentrations significantly (p<0.05) decreased in culture positive TBM-non-IRIS patients compared to comparator group

* Units are picograms per milliliter except for Cathepsin G that is presented as units per milliliter and MMP-9/TIMP-1 that is presented as a ratio

Results shown are mediator concentrations assayed with Luminex multiplex or ELISA

Statistical analyses were not performed if the medians of both comparator groups were zero

**Supplementary Table 10. P-values of correlation between cerebrospinal fluid mediators and neutrophil and lymphocyte cell counts in all TBM-IRIS and TBM-non-IRIS patients over time***

|  | **TBM diagnosis** | | | | **Starting ART** | | | | **2 weeks after starting ART †** | | | |
| --- | --- | --- | --- | --- | --- | --- | --- | --- | --- | --- | --- | --- |
|  | **TBM-IRIS** | | **TBM-non-IRIS** | | **TBM-IRIS** | | **TBM-non-IRIS** | | **TBM-IRIS** | | **TBM-non-IRIS** | |
|  | **Neutrophils Lymphocytes** | | **Neutrophils Lymphocytes** | | **Neutrophils Lymphocytes** | | **Neutrophils Lymphocytes** | | **Neutrophils Lymphocytes** | | **Neutrophils Lymphocytes** | |
| **G-CSF** | 0.71 | 0.82 | 0.36 | 0.41 | 0.86 | 0.95 | **0.03** | 0.64 | 0.86 | 0.61 | 0.12 | 0.55 |
| **GM-CSF** | 0.86 | 0.16 | 0.68 | 0.24 | 0.97 | 0.20 | 0.15 | 0.08 | 0.95 | 1.00 | **0.01** | **0.04** |
| **IFN-α2** | 0.86 | 0.87 | 0.98 | 0.82 | 0.86 | 1.00 | 0.62 | 0.28 | 0.95 | 1.00 | 0.35 | 0.32 |
| **IL-12p40** | 0.95 | 0.29 | 0.28 | 0.14 | 0.86 | 0.16 | 0.05 | 0.37 | 0.86 | 0.50 | 1.00 | 0.86 |
| **TNF** | 0.86 | 0.71 | 0.36 | 0.18 | 0.86 | 0.22 | 0.07 | 0.07 | 0.86 | 0.83 | 0.07 | 0.08 |
| **IFN-γ** | 0.86 | 0.24 | 0.68 | 0.26 | 0.86 | 0.16 | **0.03** | 0.07 | 0.95 | 0.46 | **0.03** | 0.05 |
| **IL-10** | 0.86 | 1.00 | 0.30 | 0.08 | 0.86 | 0.69 | 0.08 | 0.08 | 0.95 | 1.00 | 0.05 | 0.06 |
| **IL-1β** | 0.86 | 0.20 | 0.93 | 0.58 | 0.95 | 0.57 | 0.28 | 0.25 | 0.86 | 0.20 | 0.86 | 0.84 |
| **IL-6** | 0.86 | 0.95 | 0.10 | 0.22 | 0.86 | 0.46 | **0.03** | 0.05 | 0.86 | 0.24 | 0.05 | 0.08 |
| **IL-22** | 1.00 | 0.41 | 0.11 | 0.19 | 0.86 | 0.25 | 0.14 | 0.08 | 0.95 | 0.50 | 0.17 | 0.46 |
| **IL-18** | 0.86 | 0.85 | 0.17 | 0.22 | 0.77 | 0.25 | 0.76 | 0.20 | 0.93 | 0.45 | 0.63 | 0.49 |
| **IL-17A** | 0.86 | 0.95 | 0.06 | 0.92 | 0.95 | 1.00 | 0.63 | 0.62 | 0.86 | 1.00 | **0.03** | 0.05 |
| **CXCL1-3** | 0.86 | 0.99 | 0.76 | 0.55 | 0.86 | 0.20 | **0.03** | 0.05 | 0.86 | 0.83 | **0.04** | 0.05 |
| **CCL2** | 0.86 | 0.25 | 0.94 | 0.64 | 0.86 | 0.25 | 0.36 | 0.64 | 0.90 | 0.43 | **0.03** | 0.08 |
| **CCL3** | 0.86 | 0.82 | 0.22 | 0.07 | 0.95 | 0.99 | 0.14 | 0.31 | 0.86 | 0.56 | 0.17 | 0.28 |
| **CCL4** | 0.86 | 1.00 | 0.17 | 0.05 | 0.86 | 0.95 | 0.17 | 0.10 | 0.86 | 0.87 | 0.06 | 0.08 |
| **CXCL8** | 0.86 | 0.25 | 0.67 | 0.37 | 0.86 | 0.22 | **0.03** | 0.06 | 0.86 | 0.82 | **0.03** | 0.07 |
| **CXCL10** | 0.86 | 0.22 | 0.44 | 0.32 | 0.86 | 0.37 | 0.05 | 0.05 | 0.95 | 0.50 | 0.05 | 0.05 |
| **HNP 1-3** | 0.71 | 0.93 | 0.17 | 0.45 | 0.71 | 0.83 | 0.18 | 0.97 | 0.86 | 1.00 | 0.12 | 0.16 |
| **LL-37** | 0.86 | 0.83 | 0.10 | 0.08 | 0.86 | 0.19 | 0.77 | 0.05 | 0.95 | 0.61 | 0.17 | 0.07 |
| **Cathepsin G** | 0.86 | 0.61 | 0.61 | 0.21 | 0.95 | 0.22 | 0.44 | 0.64 | 0.86 | 0.20 | 0.56 | 0.36 |
| **Lipocalin-2** | 0.86 | 0.82 | 0.17 | 0.37 | 0.71 | 0.46 | **0.04** | 0.08 | 0.90 | 1.00 | 0.08 | 0.11 |
| **C5a** | 0.86 | 0.95 | 0.91 | 0.46 | 0.95 | 0.28 | 0.05 | 0.08 | 0.95 | 0.22 | 0.68 | 0.64 |
| **S100A8/A9** | 0.91 | 1.00 | 0.17 | 0.30 | 0.71 | 0.25 | **0.03** | 0.52 | 0.95 | 0.98 | 0.19 | 0.64 |
| **MMP-1** | 0.86 | 0.93 | 0.25 | 0.21 | 0.90 | 0.16 | 0.08 | 0.55 | 0.90 | 0.16 | 0.53 | 0.45 |
| **MMP-2** | 0.97 | 1.00 | 0.62 | 0.08 | 0.86 | 0.24 | 0.62 | **0.04** | 0.95 | 0.50 | 0.23 | 0.06 |
| **MMP-3** | 0.86 | 1.00 | 0.05 | 0.20 | 0.86 | 0.76 | 0.76 | 0.49 | 0.95 | 0.61 | 0.80 | 0.64 |
| **MMP-7** | 0.86 | 0.95 | 0.86 | 0.23 | 0.95 | 0.19 | 0.68 | **0.04** | 0.95 | 0.16 | 0.26 | 0.20 |
| **MMP-9** | 0.86 | 0.20 | 0.98 | 0.59 | 0.86 | 0.16 | **0.03** | 0.05 | 0.86 | 0.95 | **0.03** | 0.06 |
| **MMP-10** | 0.95 | 0.24 | 0.28 | 0.28 | 0.86 | 0.83 | 0.87 | 0.08 | 0.95 | 0.99 | 0.91 | 0.37 |
| **TIMP-1** | 0.90 | 0.83 | 0.07 | 0.19 | 0.86 | 0.20 | 0.31 | 0.25 | 0.86 | 0.20 | 0.41 | 0.32 |
| **TIMP-2** | 0.95 | 0.87 | 0.58 | 0.55 | 0.95 | 1.00 | 0.67 | 0.23 | 0.71 | 0.37 | 0.29 | 0.66 |

**Footnotes for Supplementary Table 10:**

*Definitions of abbreviations*: TBM, tuberculous meningitis; ART, antiretroviral therapy

Significant correlations (p<0.05) are highlighted in red

* P-values adjusted for a false discovery rate (Benjamini and Hochberg) are indicated for each correlation [5]

† For patients who developed TBM-IRIS, the “2 weeks post ART” timepoint indicates findings at time of TBM-IRIS

**Supplementary Figure 1. Boxplots of mediator concentrations over time in blood of patients who developed TBM-IRIS (red)^†^ and those who did not (blue).**

*Definitions of abbreviations:* TBM, tuberculous meningitis; ART, antiretroviral therapy

The assays’ limits of detection have been substituted for zero values. Note the left y-axis is a log 10 scale. The right y-axis indicates timepoints of sample collection. Within graphs, boxes with horizontal lines represent interquartile ranges (IQR) and medians and vertical line represent 95% confidence intervals. Data points for outliers (≥1.5 x IQR) are included. Mediators with medians equal to zero at all timepoints in both groups are not shown.

See Supplementary Tables 3, 4 and 5 for p-values of analyses between groups.

^†^ For patients who developed TBM-IRIS, the “2 weeks post ART” timepoint indicates findings at time of TBM-IRIS that developed at a median of 14 days (interquartile range, 4-20) after starting ART.

Concentrations of all mediators were measured as picograms per milliliter with the exception of Cathepsin G, which was measured in units per milliliter.

**References**

1. Bhigjee AI, Padayachee R, Paruk H, Hallwirth-Pillay KD, Marais S, Connoly C. Diagnosis of tuberculous meningitis: clinical and laboratory parameters. Int J Infect Dis **2007**; 11:348-54.

2. Meintjes G, Lawn SD, Scano F, et al. Tuberculosis-associated immune reconstitution inflammatory syndrome: case definitions for use in resource-limited settings. Lancet Infect Dis **2008**; 8:516-23.

3. Marais S, Meintjes G, Pepper DJ, et al. Frequency, severity, and prediction of tuberculous meningitis immune reconstitution inflammatory syndrome. Clin Infect Dis **2013**; 56:450-60.

4. Price RW, Spudich S. Antiretroviral therapy and central nervous system HIV type 1 infection. J Infect Dis **2008**; 197 Suppl 3:S294-306.

5. Hochberg YBY. Controlling the false discovery rate: a practical and powerful approach to multiple testing. Journal of the Royal Statistical Society Series B. **1995**; 57:289-300.

6. Hennig C. Cluster-wise assessment of cluster stability. Computational Statistics and Data Analysis **2007**; 52:258-71.
